# Early metabolic reprogramming and carbonic anhydrase IX-mediated extracellular acidification drive radiotherapy-induced glioblastoma cell dedifferentiation

**DOI:** 10.1186/s40478-025-02161-2

**Published:** 2025-11-28

**Authors:** Perrine Dahan, Tom Maillet, Laure Malric, Caroline Delmas, Vincent Lubrano, Judith Martinez-Gala, Guillaume Flandin, Amélie Aboudaram, Cécile Héliès-Toussaint, Nicolas Skuli, Sylvie Monferran, Yvan Nicaise, Clémentine Decamps, Christine Toulas, Elizabeth Cohen-Jonathan Moyal, Anthony Lemarié

**Affiliations:** 1 Université de Toulouse, INSERM, CNRS, Centre de Recherches en Cancérologie de Toulouse, Toulouse, France; 2 Oncopole Claudius Regaud, IUCT-Oncopole, Interface Department, Toulouse, France; 3 Université de Toulouse, INSERM, CNRS, Toulouse Neuro Imaging Center, Toulouse, France; 4 Neurosurgery Department, CHU de Toulouse, Toulouse, France; 5 Université de Toulouse, INRAE, ENVT, INP-Purpan, Toxalim (Research Centre in Food Toxicology), Toulouse, France; 6 Faculty of Health, Université de Toulouse, Toulouse, France; 7 Oncopole Claudius Regaud, IUCT-Oncopole, Cancer Biology Department, Molecular Oncology Division, Toulouse, France; 8 Radiation Oncology Department, IUCT-Oncopole, Oncopole Claudius Regaud, Toulouse, France

**Keywords:** Metabolic reprogramming, Radiotherapy, Glioblastoma-stem cells, Cell plasticity, Dedifferentiation, CA9, Acidification

## Abstract

Mitochondrial metabolism is enhanced in GSC compared to their differentiated progeny.

IR-induced dedifferentiation in GBM cells occurs via an early metabolic shift to OXPHOS.

IR-induced metabolic shift links CA9 induction, extracellular acidification, and stemness.

**Supplementary Information:**

The online version contains supplementary material available at 10.1186/s40478-025-02161-2.

## Abstract (structured)

**Background:**

Glioblastomas (GBM) are brain tumors with the worst prognosis despite treatment with surgery and radio/chemotherapy, emphasizing the need for new therapies and improved treatment efficacy. Previously, we showed that clinically relevant ionizing radiation (IR) doses enhance GBM cell dedifferentiation into a stem-like phenotype, increasing stemness markers, self-renewal, and tumorigenic abilities. This work focuses on identifying early mechanisms driving this plasticity, particularly metabolic adaptations, as tumor metabolism may support therapy resistance and recurrence.

**Methods:**

In this study, primary cell lines were established from GBM biopsies of several patients and cultured either in restrictive medium to form neurospheres (NS) enriched in GBM stem-like cells (GSC) or in normal medium to obtain their differentiated progenies. These differentiated GBM cells were then subjected to a short-term dedifferentiation protocol after IR (24 to 72 h) to characterize their metabolic and phenotypic properties.

**Results:**

We found that early stem marker increases after IR exposure coincide with higher oxygen consumption rate (OCR), mitochondrial ATP production, and extracellular acidification rate (ECAR). However, lactate production remained unchanged, and glucose uptake showed only a transient, nonspecific increase. This shift to oxidative mitochondrial metabolism, coupled with extracellular acidosis, promotes a stem-like phenotype and is associated with Carbonic Anhydrase IX (CA9) overexpression, an extracellular membrane protein producing H + to buffer intracellular pH. CA9 downregulation via ShRNA reduces extracellular acidification and blocks early IR-induced dedifferentiation, such as neurosphere (NS) formation and stem marker overexpression. Similarly, increased OCR and ECAR, often linked to CA9 overexpression, are observed in patient-derived GSC compared to their differentiated progenies.

**Conclusions:**

These findings highlight CA9 as a potential target to block IR-induced early dedifferentiation process as well as the specific metabolic state of GSC. Our study strengthens the therapeutic potential of CA9 inhibition to limit GBM-associated acidic extracellular environment and enhance radiotherapy efficiency by limiting tumor cell plasticity.

**Graphical abstract:**

**Supplementary Information:**

The online version contains supplementary material available at 10.1186/s40478-025-02161-2.

## Graphical abstract

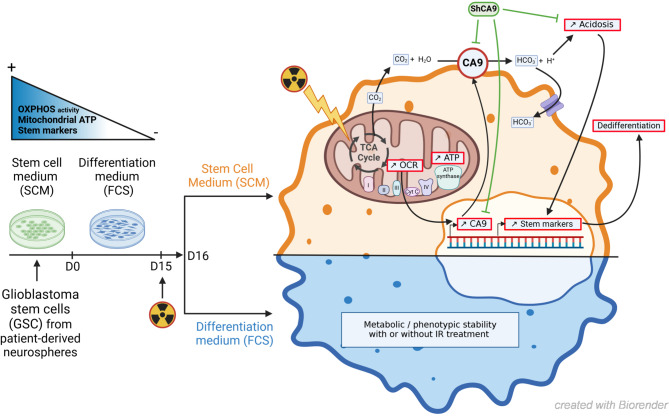

## Introduction

Glioblastomas (GBM), classified as grade 4 gliomas by the WHO [1] have a poor prognosis due to high therapy resistance. Despite standard treatment combining surgery, chemo-radiotherapy (RT) with Temozolomide (TMZ) [2] and, recently, Tumor Treating Fields (TTF), recurrence is almost inevitable, with survival ranging from 15 (without TTF) to 21 months (with TTF) [3]. This recurrence is mainly due to clonal selection of heterogeneous resistant subpopulations, particularly Glioblastoma Stem-like Cells (GSC). GSC exhibit high therapy resistance, tumorigenic potential, self-renewal and intrinsic plasticity [4]. They overexpress stem markers such as Sox2, CD133, Nestin, Olig2, A2B5, or Integrin α6 (ITGA6) [4] and can generate heterogeneous tumors in mouse models mimicking human GBM [5]. GSC therapy resistance involves enhanced various mechanisms such as a better ability to respond to DNA damages [6], to efflux drugs [7] and to activate various pathways such Notch [8] and Met [9]. Another key factor in GBM recurrence is cellular plasticity, where therapy-resistant, differentiated GBM cells could dedifferentiate into a stem-like state. We and others have shown that dedifferentiated GSC emerge in response to stresses or signals like IR [10], hypoxia [11], TMZ [12, 13], HGF [14], bFGF [15], EGR/EGFR/EGR1 [16] or CMV-infection [17], expanding the resistant, invasive and tumorigenic cancer stem cell (CSC) pool. Similar dedifferentiation occurs in pancreatic [18], lung [19] or colorectal [20] cancers, in response to different stimuli. However, in GBM, the mechanisms driving and regulating this process remain poorly understood.

Understanding this therapy-induced phenotypic plasticity in GBM could reveal new therapeutic targets. We previously identified the anti-apoptotic protein survivin as a mediator of IR-induced dedifferentiation in vitro [10]. This study explores the early metabolic mechanisms driving IR-induced reprogramming. Since metabolic changes often drive cancer cell adaptation and plasticity [21] and support key GSC traits like self-renewal [22], we investigated the energetic metabolic alterations underlying IR-induced plasticity in GBM cells. Targeting metabolism has shown therapeutic potential in cancers [23], including GBM, where metabolic changes contribute to chemo/radioresistance [24–26]. We previously showed that Metformin, by inhibiting mitochondrial respiration and activating AMPK, shifts metabolism toward glycolysis in GBM cell lines, reducing tumor growth in mice xenografts and enhancing TMZ and IR sensitivity in vitro [27]. IR effects on metabolism are complex. A study on differentiated GBM cell lines showed that IR reduces oxidative phosphorylation (OXPHOS) in favor of glycolysis, and that inhibiting glycolysis improves survival in treated mice treated with IR [26]. Conversely, other studies suggest IR-exposed GSC enhance their repair capacity of DNA breaks and lipid peroxidation via metabolic adaptations, enriching resistant clones which rely less on glucose and can maintain ATP production primarily through OXPHOS [28]. GSC were also shown to use alternative fuels like glutamine [29] and fatty acid [30] to sustain the Tricarboxylic Acid cycle (TCA) and OXPHOS. Notably, glutamine metabolism has been linked to GSC radioresistance [31]. These findings suggest that GSC and differentiated GBM cells exhibit distinct metabolic responses to therapy, both contributing to radioresistance and tumorigenesis. We hypothesize that low-dose IR triggers energetic metabolic reprogramming, driving GBM dedifferentiation into a stem-like phenotype. Studies support this by linking specific metabolic states to GBM cell differentiation and tumorigenicity [32–34]. Additionally, metabolic reprogramming may alter the tumor microenvironment, inducing extracellular acidosis via pH-regulating enzymes and carriers, like Carbonic Anhydrases (CA), Na + /H + exchangers (NHE) and Vacuolar H^+^-ATPase (V-ATPase) [35]. This extracellular pH (pHe) drop was shown to promote various cancer processes [36], such as therapy resistance [37, 38], invasion [39] and stemness maintenance in GBM cells [40, 41].

Our data show that IR-induced dedifferentiation in primary GBM cells, previously highlighted by us [10], is driven by an early metabolic shift with increased O₂ consumption and extracellular acidification. This CA9-driven acidosis after clinically relevant IR doses promotes phenotypic plasticity toward a stem-like state in GBM cells. We demonstrate here for the first time that IR, a key GBM therapy, promotes early metabolic and subsequent phenotypic reprogramming, potentially expanding the GSC pool and driving rapid tumor recurrence.

## Materials and methods

### GSC isolation and cell culture

C1, D1, G and I GSC patient cell lines were established as described [10]. SRA5, SRB1 and SRC3 GSC patient cell lines were derived from the STEMRI clinical trial (NCT01872221) [42, 43]. Forced differentiation and IR-induced dedifferentiation (3 Gy) followed our published protocol [10], with a shorter 1-day interval before medium replacement with either FCS-differentiating medium or dedifferentiation-permissive Stem Cell Medium (SCM). See details in Supplementary Methods.

### Oxygen consumption rate (OCR) and extracellular acidification rate (ECAR) measurements (Seahorse XF assay)

OCR and ECAR were measured in real time using Seahorse XF24/XF96e (Agilent) as described by us and others [27, 34], using MitoStress (Agilent) kit (see Supplementary Methods).

### ATP measurement

ATP levels were assessed using the Cell Titer-Glo 2.0 assay (Promega) after appropriate treatments to determine total, glycolytic and mitochondrial ATP, as described [27] (see Supplementary Methods).

### Glucose uptake assay

Glucose uptake assay followed a previous study [44] on GDC cells plated in 12-well plates (7.5 × 10^3^ cells/cm^2^) before IR and medium change 1 day post-IR. Cells were washed with serum-free DMEM low glucose, pre-incubated for 4h at 37 °C, washed with Krebs–Ringer-Bicarbonate (KRB) buffer and incubated 30min with 100 nM insulin (or cytochalasin B as control, Merck). Glucose uptake was initiated by adding [^3^H] 2-Deoxy-D-glucose (1 μCi/mL, PerkinElmer) in 0.1 mM D-glucose and incubating 10min at 37 °C. After washing with ice-cold KRB, cells were solubilized in 0.1N NaOH. Half of each well was transferred into scintillation vials with 10mL of scintillation cocktail, Ultima Gold LLT (PerkinElmer). Radioactivity was measured by scintillation counting (Hewlett Packard counter), and protein content normalized.

### Lactate measurement

Lactate levels were measured using the Lactate Colorimetric Assay kit (Sigma-Aldrich) as described [27]. Supernatants from each condition were analyzed in triplicate, with protein normalization.

### shRNA transfection and selection

GDC from D1, G, I and SRC3 patients were plated in 6-well plates (700 000 cells/well) and transfected, using Fugene HD (Promega), with 3µg of plasmids encoding either specific shRNA for CA9 (ShCA9-1 and 4) or unspecific shCTR (Qiagen, Supplementary Table S1). After five days, transfected cells were selected for at least 3 weeks in FCS medium with 0.8 mg/mL G418 (Merck).

### Quantitative real-time RT-PCR (qPCR)

qPCR was performed as described [10] (see Supplementary Methods). Some experiments used commercial RNA (Supplementary Table S2).

### Western blotting and flow cytometry

Western-blot and flow-cytometry were conducted as shown [10] (see Supplementary Methods)*.*

### Neurospheres generation assay

Limiting dilution assays were conducted as described [45] (see Supplementary Methods)*.*

#### RNA-sequencing (RNA-seq) analysis

RNA-seq data were downloaded from NCBI Gene Expression Omnibus (GEO) GSE54791, originating from a study by Suva et al. comparing three different human GSC cell lines (MGG4, MGG6 and MGG8) and their matched differentiated progeny (GDC) [46]. Each cell line was analyzed in triplicate condition. Briefly, the counting table (in CPM) has been downloaded from GSE54791 and only genes with sufficient information (more than 10 counts across samples) were retained. The data were then log-normalized (log2(CPM + 1)) and differential analysis was performed with limma [47], taking into account biological replicates and cell types. The p-values were corrected for multiple testing using the method of Benjamini & Hochberg (BH).

Molecular subtype of the GSC cell lines used in the present study was determined according to Wang classification [48], using the Gliovis data portal and the SubtypeME tool (http://gliovis.bioinfo.cnio.es) [49], in order to classify our GSC into three tumor-specific subtypes: proneural (PN), mesenchymal (Mes), and classical (CL). Affymetrix array datasets (for C1, D1 and I GSC) and RNA-seq datasets (for SRA5, SRB1 and SRC3 GSC), obtained from our previous publications ([50] and [51], respectively), were used to subtype our different GSC. For SRA5, SRB1 and SRC3 GSC, we also determined in a previous publication the molecular subtypes of the patients’ biopsies from whom these GSC were derived [42]. These subtypes are described in Supplementary Table S3 and illustrate the molecular heterogeneity of the GSC cell lines used in this study.

#### Statistical analysis

Data are means ± S.E.M. ≥ 3 independent experiments. Significance (*P < 0.05, **P < 0.01 and ***P < 0.001) was assessed using the Student *t*-test.

## Results

### Early stem marker expression after IR in GBM Cells subjected to the dedifferentiation protocol

**Fig. 1:**
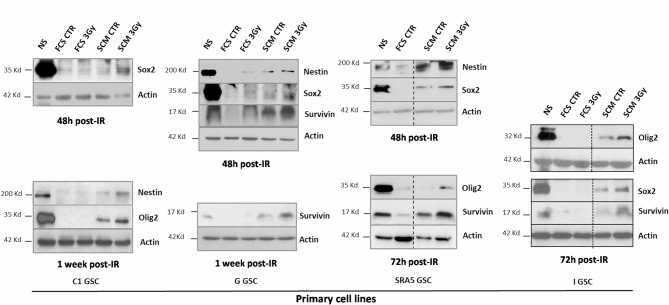
Early overexpression of stemness markers during irradiation-induced dedifferentiation. Differentiated GBM cells from 4 patient cell lines (C1, G, I and SRA5) treated or not by a 3 Gy irradiation (IR) and placed 1 day after in either FCS or SCM medium for short-term culture (2 to 7 days post-IR) were analyzed by western blotting for stem marker (Olig2, Sox2 and Nestin) and Survivin expression. Protein expression levels in GSC-enriched NS (neurosphere) parental cells were shown as a control for the stem condition. Equal gel loading and transfer efficiency were checked with anti-actin antibody. Blots are representative of at least three independent experiments

To investigate early mechanisms which could initiate and drive the long-term dedifferentiation induced in GBM cells at 30 days after IR exposition at least [10], we analyzed several stem cell transcription factors (SCTF) and stem markers from 48h to one week post-IR. Olig2, Sox2, and Nestin, highly expressed in parental GSC lines, were specifically upregulated in irradiated cells in stem medium (SCM 3Gy) as early as 48–72h post-IR, with little or no expression in non-irradiated controls (SCM CTR) (Fig. 1). This early SCTF induction, observed in four primary GBM cell lines (C1, G, I and SRA5), correlates with IR-induced long-term dedifferentiation in the same cell lines, enhancing self-renewal and tumorigenesis [10]. As in the long-term protocol [10], IR did not modulate stem marker expression in forced differentiation conditions (FCS). Additionally, survivin, an anti-apoptotic protein mediating IR-induced dedifferentiation [10], was upregulated alongside SCTF as early as 48h post-IR (Fig. 1). These findings suggest that both SCTF and survivin are rapidly induced in permissive SCM conditions and maintained throughout the dedifferentiation process, promoting the phenotypic transition to a stem-like state.

### Metabolic disparity between GSC-enriched NS and their differentiated progeny

**Fig. 2:**
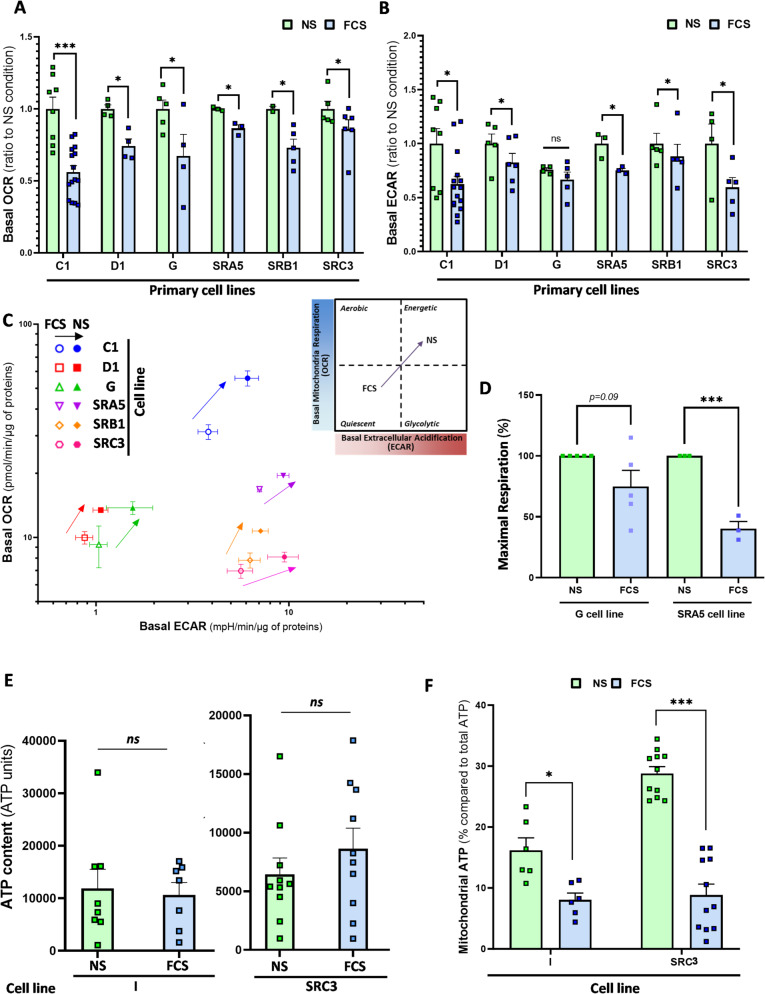
Differences in the metabolic state of GSC and their differentiated progeny. GSC-enriched NS cell lines isolated from 7 patient tumors (C1, D1, G, I, SRA5, SRB1, SRC3) were kept in SCM medium (NS) or allowed to differentiate as adherent GBM cells for at least 15 days in FCS medium (FCS). **A**–**C** Seahorse analysis of **A** basal Oxygen Consumption Rate (OCR), **B** Extracellular Acidification Rate (ECAR) and **C** energy phenograms of mean OCR and ECAR, after normalization to total protein content for the indicated primary GBM cell lines in FSC or NS culture condition. In **A** & **B**, results are expressed as ratios to each NS condition. **D** Seahorse analysis of Maximal Respiration in G and SRA5 primary cell lines in FSC or NS culture condition, after normalization to total protein content. Results are expressed as percentages relative to NS condition. **E** Total intracellular ATP content in either NS or FCS condition in the indicated primary GBM cell lines. **F** Percentage of mitochondrial ATP was determined in the indicated primary GBM cell lines. **A**–**F** Shown are the means ± S.E.M. of at least three independent experiments  *P < 0.05, ***P < 0.001 compared with the NS condition. *ns* non-significant

To uncover early mechanisms driving short-term adaptation in GBM cells to subtoxic IR dose and SCTF overexpression at 48h post-IR, we analyzed global metabolic pathways supporting ATP production and anaplerotic biosynthesis (lipids, amino acids), crucial for proliferation, stress repair, and survival in GBM [52]. While differentiated GBM cells favor glycolysis and the Warburg effect, GSC rely more on oxidative phosphorylation (OXPHOS), exhibit lower glucose dependency, and display higher oxygen consumption rates (OCR) in CD133 + GSC [32–34]. Using Seahorse system, we measured OCR and ECAR (extracellular acidification rate) in six NS cell lines (C1, D1, G, SRA5, SRB1, SRC3) and their differentiated progeny after at least 15 days of forced differentiation [10]. NS showed significantly higher OCR and ECAR than their differentiated counterparts, reflecting a more energetic phenotype with an increased mobilization of the O_2_-consuming/CO_2_-producing mitochondrial metabolism for ATP production [53], and a higher maximal respiration (MR) (Fig. 2A–D). Indeed, an elevated ECAR, typically associated with glycolysis via H^+^/lactate extrusion by monocarboxylate transporters (MCT), may also result from a high CO_2_ production due to a fast-cycling TCA cycle. This CO_2_ is then expelled from the cell and converted into bicarbonates through a H^+^-generating reaction catalyzed by extracellular carbonic anhydrases (CA) [35, 54]. Thus, the increased ECAR in NS likely reflects enhanced mitochondrial metabolism, as indicated by higher OCR and MR levels.

Despite this higher energetic state, total ATP content was similar between GSC (I, SRC3) and their differentiated counterparts (Fig. 2E), suggesting that GSC and GDC may produce a similar ATP level but through distinct energetic pathways. It was indeed shown that this ATP production in NS and FCS-differentiated cells has not the same origin, either through OXPHOS or aerobic glycolysis, respectively [32]. Selective inhibition of these pathways (see Materials & Methods) confirmed that NS cells from I and SRC3 patients rely more on mitochondria and OXPHOS for ATP production than their differentiated progeny (Fig. 2F). These results suggest that metabolic differences are linked to GBM cell differentiation status and may emerge during IR-induced plasticity.

### Early metabolic shift in GBM cells during the first steps of the radio-induced dedifferentiation

**Fig. 3:**
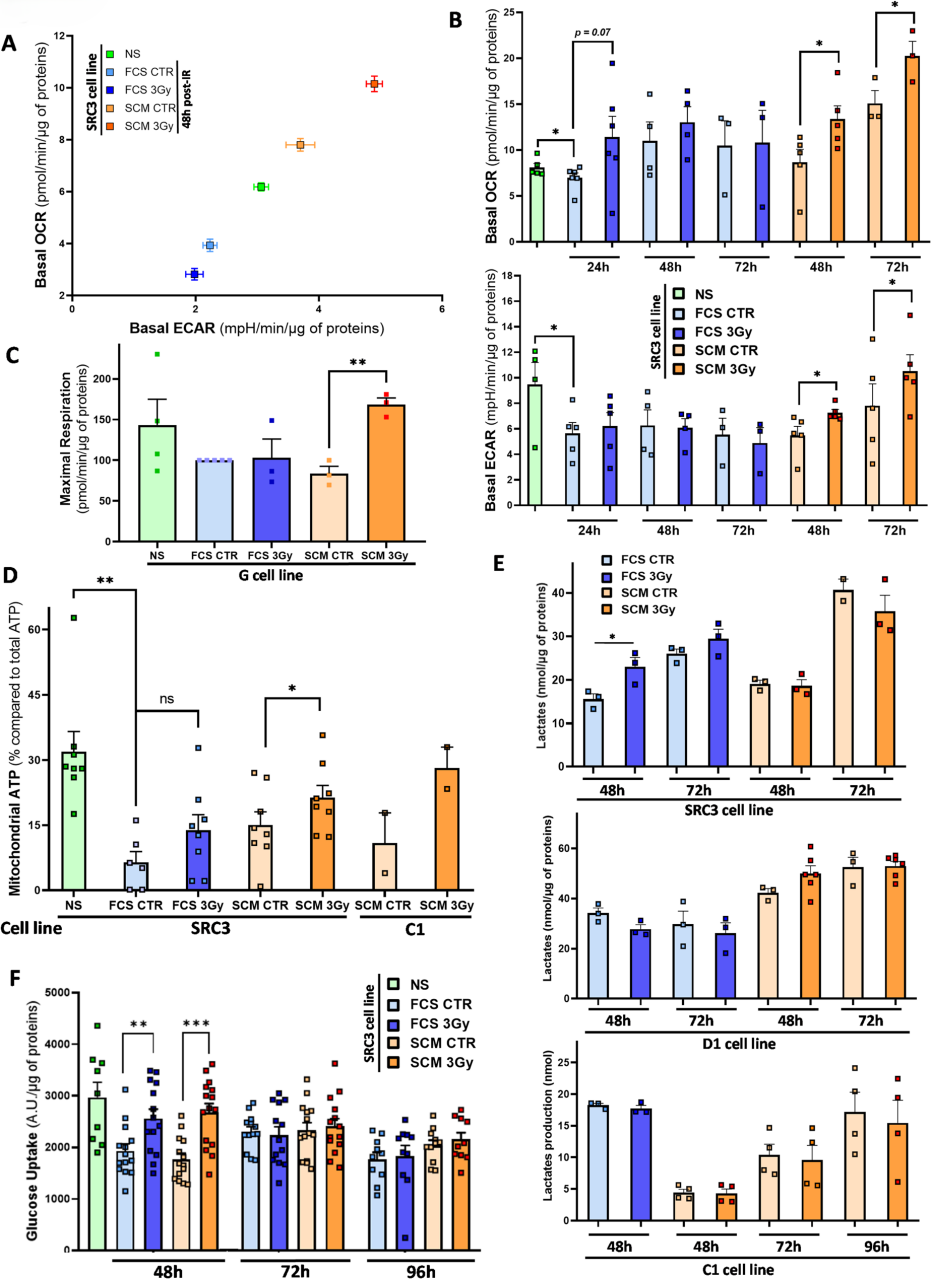
Early energetic metabolic shift during IR-induced dedifferentiation. Differentiated GBM cells from the indicated patient cell lines (C1, D1, G, SRC3) treated or not by a 3 Gy irradiation and placed 1 day after in either FCS or SCM medium for short-term culture (24h to 96h post-IR) were analyzed for different metabolic parameters. **A** Representative energy phenogram of OCR and ECAR shift during IR-induced dedifferentiation at 48h post-IR in SRC3 cells either in FSC or SCM condition. Mean OCR and ECAR are shown after normalization to total protein content. **B** Seahorse analysis of basal OCR and basal ECAR of SRC3 cells during the dedifferentiation protocol in either FCS or SCM condition at 24h, 48h and 72h post-IR after normalization to total protein content. NS condition was shown as a control for the stem condition. **C** Seahorse analysis of Maximal Respiration in G primary cell line in FSC or SCM culture condition at 72h post-IR, after normalization to total protein content. Results are expressed as percentages relative to FCS CTR condition. **D** The proportion of mitochondrial ATP (in % compared to total ATP) was determined in SRC3 and C1 cells subjected to the dedifferentiation protocol at 72h post-IR. SRC3 NS condition was shown as control for the stem condition. **E** Lactate content in cell supernatants of SRC3, D1 and C1 cells subjected to the dedifferentiation protocol (48h to 96h post-IR), after normalization to total protein content. **F** Glucose uptake capacity in SRC3 cells subjected to the dedifferentiation protocol at 48h, 72h and 96h post-IR, after normalization to total protein content. **B**–**F** Shown are the means ± S.E.M. of at least three independent experiments, except for C1 cells in (**D**) (n = 2).  * *P* < 0.05,  **P < 0.01, ***P < 0.001 compared with the indicated condition. *ns* non-significant

To explore the role of metabolic differences between parental NS and their differentiated progeny in early IR-driven dedifferentiation, Seahorse analysis was performed on five primary cell lines exposed to IR for 24–72h in FCS or SCM medium. In all cell lines (D1, G, SRA5, SRB1 and SRC3), OCR and ECAR specifically and significantly increased after IR in SCM at 48h or 72h post-IR (Fig. 3A, B and Supplementary Figure S1). In SRC3 cells, a kinetic analysis showed OCR enhancement in permissive SCM medium at 48h post-IR, further increasing at 72h, compared to non-irradiated cells, along with a similar rise in ECAR. In contrast, no significant OCR or ECAR increase was observed in FCS at any time point (Fig. 3B). Similar expression patterns, illustrated by representative energy phenograms, were observed in the four other cell lines at 48h or 72h post-IR (Supplementary Figure S1). In G cells, increased OCR correlated with higher mitochondrial MR levels in SCM post-IR (Fig. 3C). These findings suggest that IR-induced dedifferentiation occurs specifically in cells undergoing an early and sustained metabolic shift, characterized by increased O_2_ consumption and extracellular acidification.

To analyze glycolysis and OXPHOS processes during early dedifferentiation, ATP sources were examined at 72h post-IR. SRC3 cells showed a significant increase in mitochondrial ATP in SCM 3Gy condition, with a similar trend in C1 cells, but no changes in FCS conditions (Fig. 3D). No changes in total ATP levels were observed in SRC3, C1, and I cell lines under the same culture conditions, with or without IR (Supplementary Figure S2). This confirms a shift to OXPHOS in response to IR specifically in permissive SCM medium. Lactate, a product of aerobic cytosolic glycolysis, was also quantified in supernatants during in the first dedifferentiation steps. In three cell lines (SRC3, C1, D1), IR did not induce lactate increase in permissive SCM at 48 or 72h post-IR. In FCS medium, only SRC3 cells showed a brief lactate overproduction at 48h post-IR (Fig. 3E). We also checked this lactate production all along the long-term dedifferentiation protocol in G cells and did not observe any lactate increase after IR in either SCM or FCS medium (Supplementary Figure S3). Thus, the significant ECAR increase observed at 48/72h post-IR in SCM does not appear to result from increased lactate production via aerobic glycolysis.

To assess whether IR-induced mitochondrial oxidative metabolism is fueled by glucose via pyruvate synthesis, TCA cycle and subsequent OXPHOS, we quantified glucose uptake at 48h, 72h, and 96h post-IR in SRC3 cells (Fig. 3F). A transient glucose uptake increase was observed at 48h in both FCS and SCM conditions after IR but it disappeared at 72h and 96h. Given the sustained OCR increase at 72h post-IR in SCM but not in FCS condition, this brief glucose uptake rise likely does not drive OXPHOS activation during dedifferentiation. Instead, it may serve as an early defense mechanism for repair and survival following IR-induced damages, independently of the culture condition. This suggests that other substrates, such as glutamine or fatty acids, contribute to IR-induced OXPHOS reprogramming and plasticity.

### Early overexpression of carbonic anhydrase IX (CA9) during IR-induced dedifferentiation

**Fig. 4:**
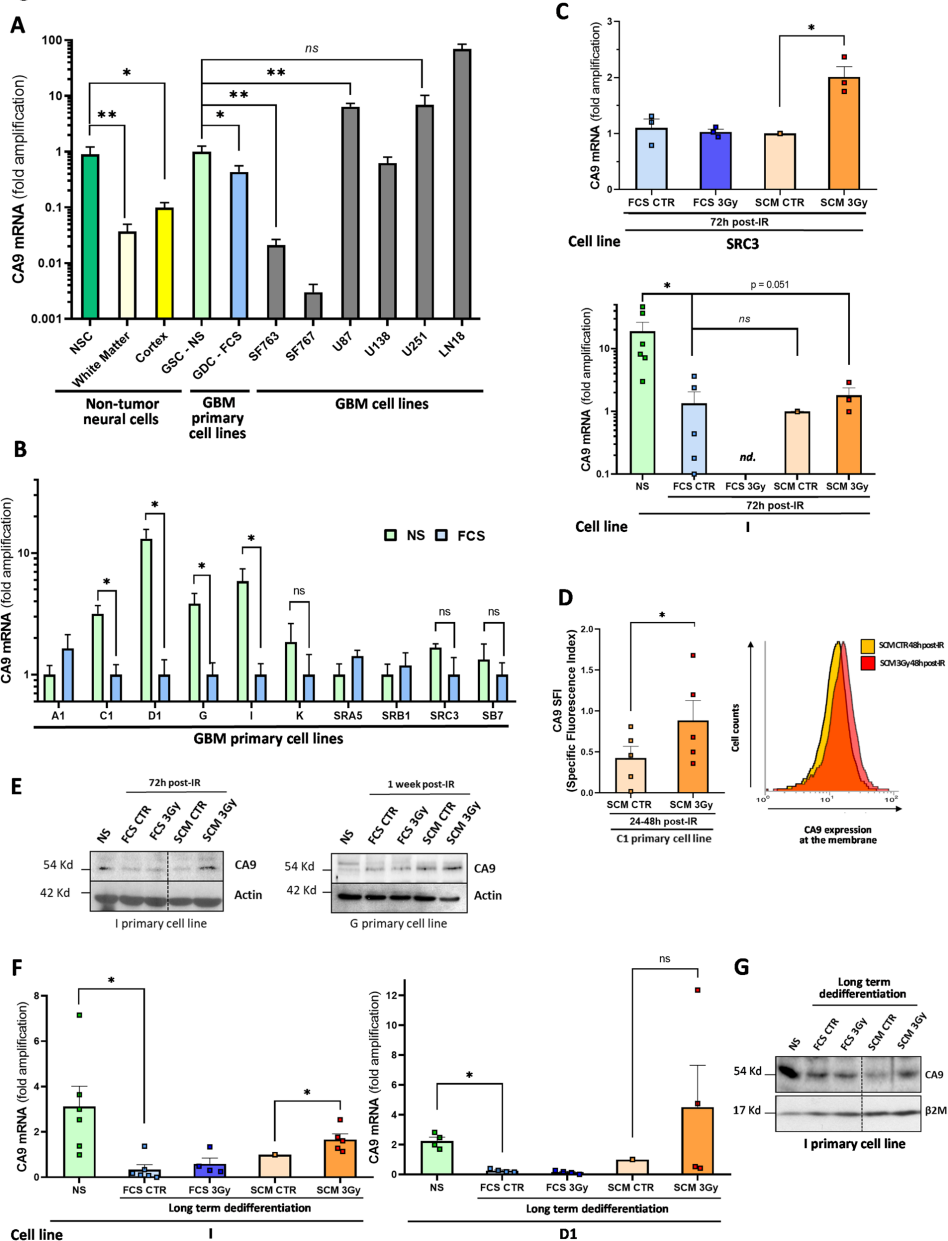
Carbonic anhydrase IX (CA9) overexpression in GSC and during IR-induced dedifferentiation. **A** mRNA expression levels of CA9 in NSC (mean of 3 different samples), adult brain cortex (mean of 9 different samples), adult normal white matter (mean of 1 sample analyzed in triplicate), human GSC-enriched NS (mean of 10 different patient GSC cell lines) and their differentiated progeny (FSC, mean of 10 different cell lines) and six conventional human GBM cell lines (SF763, SF767, U87, U138, U251 and LN18, with at least 3 independent samples per cell line, except for SF767 and U138 (n = 2)). Shown are the fold amplifications relative to the indicated condition expressed as means ± S.E.M. **B** mRNA expression level of CA9 in GSC-enriched NS cell lines and in their differentiated counterparts kept at least 15 days in FCS medium (FCS). Shown are fold inductions relative to the FCS condition expressed as means ± S.E.M. of at least three independent experiments. **C** mRNA expression level of CA9 at 72h post-IR during the dedifferentiation process in SRC3 and I cell lines. Shown are the fold inductions relative to the indicated condition expressed as means ± S.E.M. of at least three independent experiments. *nd*: not determined. **D** FACS analysis of CA9 at the membrane of C1 cells at 24-48h post-IR (left panel). Representative FACS plot overlay (SCM CTR versus SCM 3 Gy) was depicted (right panel). Shown are the means ± S.E.M. of Specific Fluorescence Index (SFI) values for five independent experiments. **E** Western blotting analysis of CA9 expression in I or G cell lines at the indicated time post-IR during the early dedifferentiation process. NS conditions were shown as controls for the stem condition. Equal gel loading and transfer efficiency were checked with anti-actin antibody. Blots are representative of three independent experiments. **F**,** G** Expression of CA9 in I or D1 cell lines during the long-term dedifferentiation process. mRNA **F** and protein **G** expression levels of CA9 were analyzed at the end (30 days post-IR) of the dedifferentiation protocol. NS conditions were shown as controls for the stem condition. Quantitative PCR results are expressed as fold inductions relative to the SCM CTR condition and are shown as means ± S.E.M. of at least three independent experiments. For blots, equal gel loading and transfer efficiency were checked with an anti-β2M antibody. Blots are representative of three independent experiments.** A**–**D**, ** F** *P < 0.05,  **P < 0.01, ***P < 0.001 compared with the indicated condition. *ns* non-significant

ECAR increased over time after IR specifically in permissive SCM medium (Fig. 3B). Since lactate level remained stable across all conditions (Fig. 3E), lactate/H⁺ extrusion is likely not the main driver of extracellular acidification. To explore alternative mechanisms behind extracellular acidosis, we examined another source of acidosis in tumor cells, i.e. CO_2_ production via TCA cycle and mitochondrial metabolism [35]. Indeed, CO_2_ can be expelled into the extracellular microenvironment, generating protons and bicarbonate ions through Carbonic Anhydrases, with HCO₃⁻ re-entering cells to buffer intracellular pH and maintain cellular function homeostasis. Among these enzymes, Carbonic Anhydrase IX (CA9) is highly expressed in GBM biopsies and linked to poor prognosis in GBM patients [55, 56]. As CA9 is also associated with stemness maintenance in breast and GBM CSC models [57–59], we analyzed it expression in normal adult brain cells (cortex/white matter), Neural Stem cells (NSC), various conventional GBM cell lines (SF763, SF767, U87, U138, U251 & LN18) and our patient-derived NS and their differentiated progenies (Fig. 4A). Normal adult brain cells expressed very low CA9 mRNA levels compared to GBM samples (except SF763/SF767 cell lines). Additionally, a pool of 10 GSC-enriched NS cell lines derived from patient resections exhibited significantly higher CA9 expression than their differentiated progeny. NSC also overexpressed CA9 compared to normal adult brain cells, at levels similar to GSC. Comparing CA9 mRNA expression between NS and FCS-differentiated conditions for each NS cell line, we confirmed that 4/10 NS lines (C1, D1, G, I) significantly overexpressed CA9, with a similar trend in one additional line (K). However, the remaining five NS lines showed similar CA9 levels between differentiation states (Fig. 4B). Since both CA9-overexpressing and non-overexpressing NS cells showed increased ECAR than differentiated cells, basal CA9 expression alone may not explain extracellular acidification in all GSC lines. In some GSC cultures, another extracellular acidifying system may complement or surpass CA9 to stabilize the metabolic stem state.

However, regardless of CA9 overexpression in GSC compared to their differentiated progeny, we observed a specific upregulation of CA9 mRNA during the early dedifferentiation stages in SRC3 at 72h post-IR in permissive SCM, with a similar trend in I cells (Fig. 4C). This was confirmed at the protein level in C1, G, and I cell lines through western-blot or FACS analysis (Fig. 4D, E). Moreover, CA9 overexpression in response to IR in SCM persisted throughout long-term dedifferentiation, as the same expression pattern was observed at both mRNA (I and D1 cells) and protein levels (I cells) 30 days post-IR (Fig. 4F, G). Collectively, these data suggest that IR-induced CA9 overexpression in SCM is linked to (i) an oxidative metabolic shift and (ii) extracellular acidification in GBM cells. This led us to investigate whether CA9 plays a direct role in the dedifferentiation process by promoting phenotypic plasticity toward a stem-like state via CA9-dependent extracellular acidosis.

### Role of early CA9 overexpression in IR-induced dedifferentiation of GBM cells

**Fig. 5:**
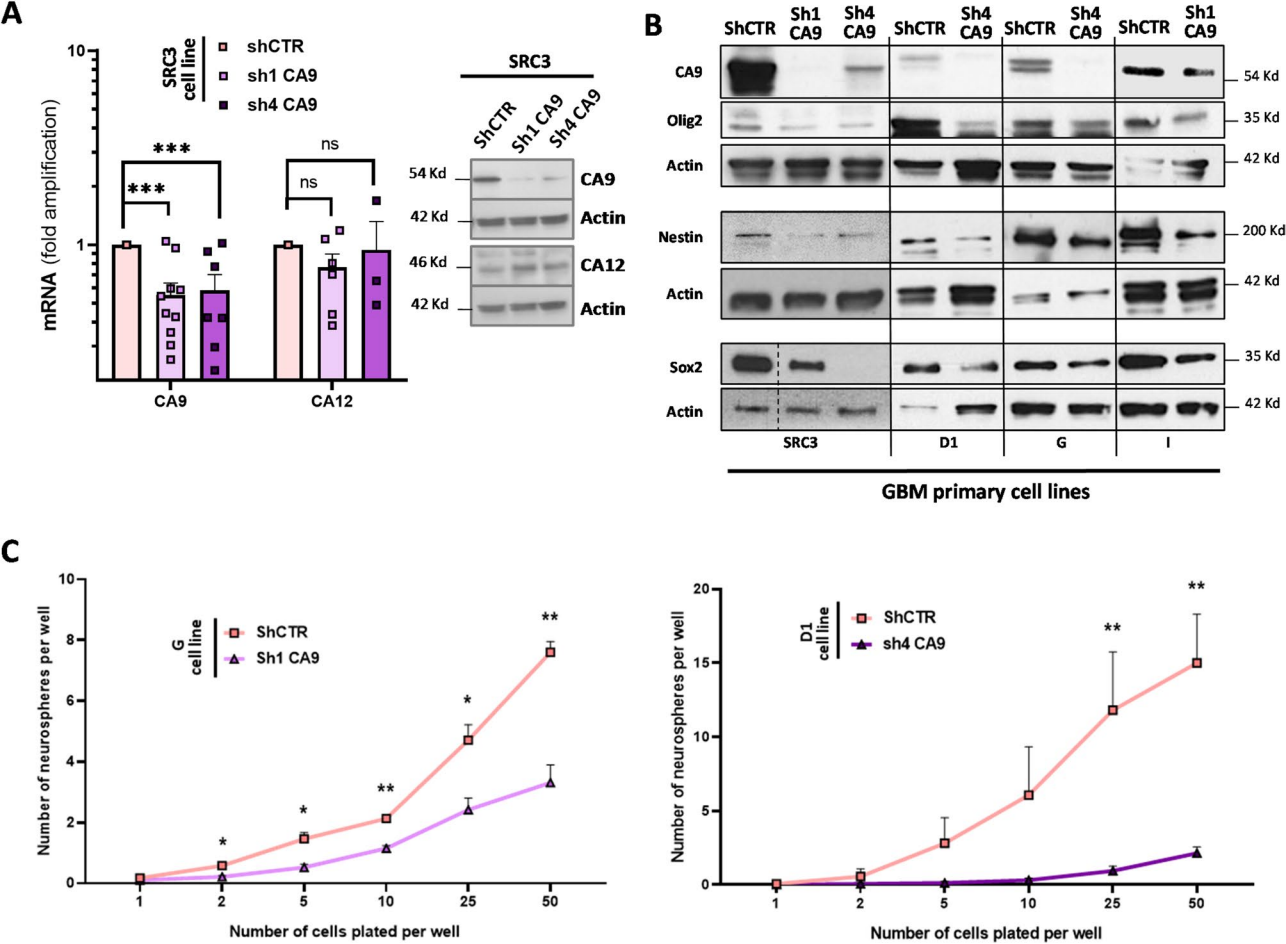
Role of CA9 in the stemness maintenance.** A**–**C** SRC3, D1, G or I cells were transfected by either shCTR (non-specific control) or two different shRNA against CA9 (sh1-CA9 and sh4-CA9). Transfected cells were then selected in G418-containing SCM medium in order to check CA9 expression by real-time qPCR and Western-blotting **A**, as well as to analyze the effects of CA9 knockdown on stem markers expression **B** and the neurosphere forming ability through limiting dilution assay **C**. **A** mRNA and protein expression levels of CA9 and CA12 in stably transfected SRC3 cells expressing shCTR, sh1-CA9 or sh4-CA9. Shown are the fold inductions relative to the shCTR condition expressed as means ± S.E.M. of at least three independent experiments. *ns* non-significant, ***P < 0.001. For Western blotting, equal gel loading and transfer efficiency were checked with anti-actin antibody. Blots are representative of at least three independent experiments.** B** Western blotting analysis of CA9, Olig2, Nestin and Sox2 in stably transfected SRC3, D1, G and I cells expressing shCTR, sh1-CA9 or sh4-CA9. Blots are representative of at least three independent experiments for SRC3 and two experiments for D1, G and I cells.** C** Neurosphere (NS) forming assay performed with stably-transfected G (left panel) or D1 (right panel) cells expressing shCTR, sh1-CA9 (G cells) or sh4-CA9 (D1 cells). Assays were performed in limiting dilution conditions as described in *Materials and Methods*. Shown are means ± S.E.M of NS number per well of three independent experiments for G cells and one experiment for D1 cells (N = 16 technical replicates). **A, C**  *P < 0.05,  **P < 0.01, ***P < 0.001 compared with the indicated condition. *ns*: non-significant

To investigate CA9’s role in IR-driven early dedifferentiation in GBM cells, we stably suppressed its expression in patient-derived differentiated cells. SRC3 cells were transfected with two distinct CA9-targeting shRNAs (Sh1 CA9 and Sh4 CA9) and selected in G418 for at least three weeks. Both shRNAs effectively downregulated CA9 at the mRNA and protein levels, confirmed by western-blot and flow cytometry (Fig. 5A, Supplementary Figure S4A), compared to shControl (shCTR). To exclude compensation by CA12, another CA with lower catalytic efficiency and also expressed at the extracellular membrane side [60], we analyzed its expression in CA9-deficient cells and found no significant modulation, thus excluding the possibility of compensation for CA9 loss (Fig. 5A). Since CA9 is linked to stemness in breast and GBM CSC [57–59], we performed Western-Blot analysis of stem markers Olig2, Sox2, and Nestin in SRC3, D1, G, and I GSC following CA9 inhibition via shRNA. All cell lines showed decreased stem-like markers after CA9 inhibition, reinforcing CA9’s role in stemness (Fig. 5B). Similar trends were observed at the mRNA level, with decreased Olig2/Sox2 and increased GFAP/TUJ1 differentiation markers in G or SRC3 cells transfected with CA9-targeting shRNA (Supplementary Figure S4B, C). Finally, extreme limiting dilution assays revealed reduced NS formation in CA9-knockdown D1 and G GSC, indicating impaired self-renewal (Fig. 5C). These findings demonstrate that stable CA9 inhibition via shRNA in GSC is not compensated by CA12 and significantly disrupts GSC stemness maintenance.

### Role of CA9 in extracellular acidosis and cell plasticity during IR-induced dedifferentiation

**Fig. 6:**
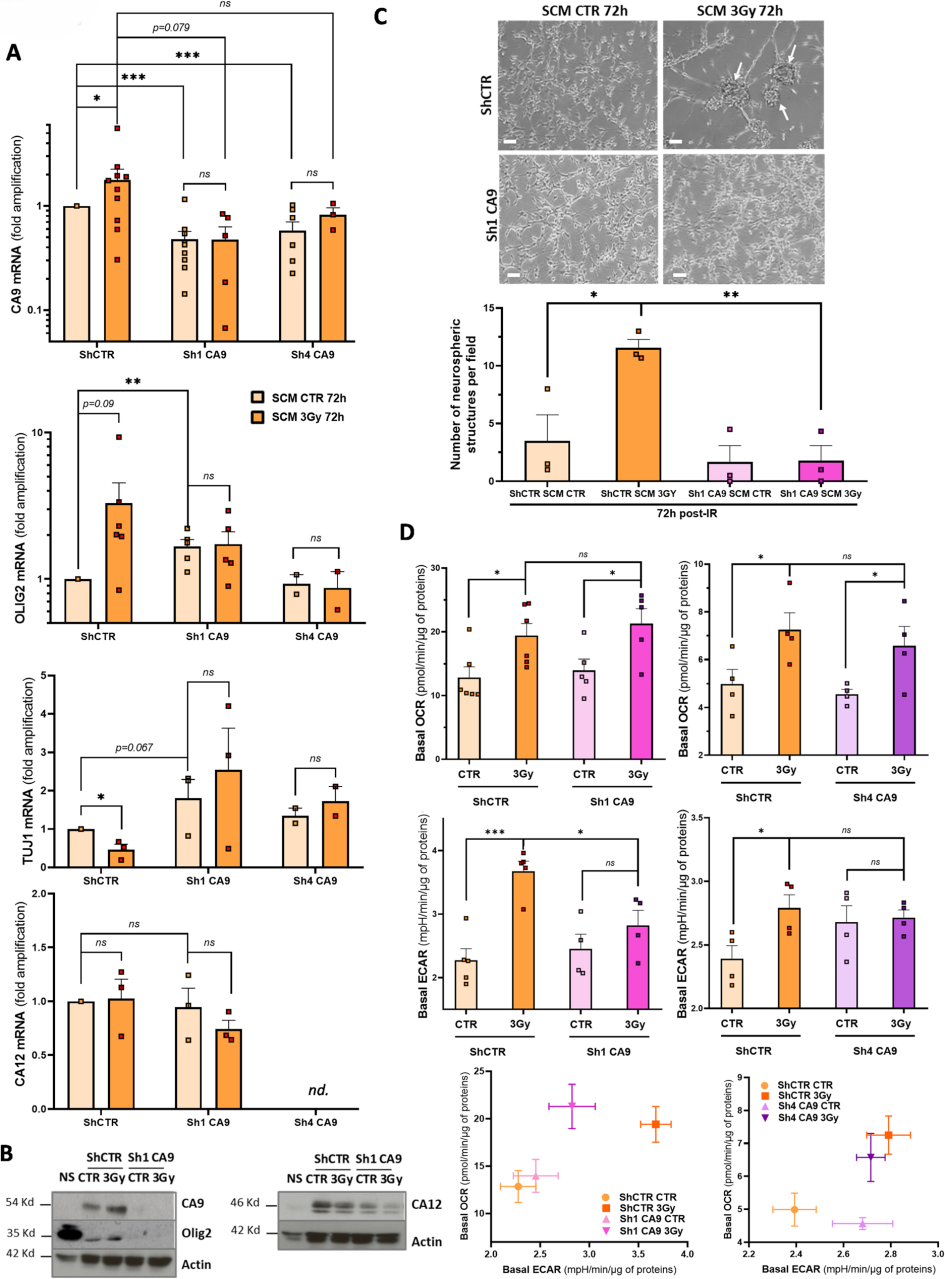
Role of CA9 in the early steps of IR-induced dedifferentiation process and metabolic alterations induced by IR in SCM medium. Differentiated stably-transfected SRC3 cells with either shCTR, sh1-CA9 or sh4-CA9 were treated or not by a 3Gy irradiation and subjected to the short-term dedifferentiation protocol in SCM medium. **A** mRNA expression level of CA9, Olig2 (stem marker), TUJ1 (differentiation marker) and CA12 in stably-transfected SRC3 cells expressing shCTR, sh1-CA9 or sh4-CA9 maintained in SCM medium 72h post-IR. Results are expressed as the means ± S.E.M. fold inductions relative to the shCTR cells in SCM CTR condition of at least three independent experiments. **B** Western blotting analysis of CA9, CA12, Olig2 in stably transfected SRC3 cells expressing shCTR or sh1-CA9 subjected to the dedifferentiation protocol in SCM medium at 72h post-IR. Equal gel loading and transfer efficiency were checked with anti-actin antibody. Blots are representative of three independent experiments.** C** Phase-contrast photomicrographs (upper panel) of SRC3 cells stably expressing shCTR or sh1-CA9 subjected to the dedifferentiation protocol in SCM medium at 72h post-IR (Original magnification: × 10, scale bar: 6 μm). On the lower panel, shown are the quantifications of the number of neurospherical structures (arrows) counted per well, expressed as means ± S.E.M of at least three independent experiments. **D** Seahorse analysis of basal OCR (upper panel), basal ECAR (middle panel) and related energy phenograms (lower panel) in stably transfected SRC3 cells expressing shCTR, sh1-CA9 or sh4-CA9 subjected to the dedifferentiation protocol in SCM medium at 48h post-IR. Quantifications of OCR and ECAR variations are expressed as the means ± S.E.M. of at least four independent experiments, after normalization to total protein content. **A**, **C**, **D**  *P < 0.05, **P < 0.01,  ***P < 0.001 compared with the indicated condition. *ns* non-significant, *nd* non-determined

To assess CA9's role in early molecular changes during IR-induced dedifferentiation, transfected differentiated cells underwent the in vitro dedifferentiation protocol in SCM and were analyzed at 72h post-IR. Stem (Olig2) and differentiation (TUJ1) markers were examined at the mRNA and protein levels to confirm CA9’s contribution to stem phenotype acquisition (Fig. 6A, B). CA9-targeting shRNAs effectively reduced basal and IR-induced CA9 expression at 72h post-IR in SRC3 cells, without affecting CA12, which remained unchanged after IR. Notably, CA9 knockdown completely prevented IR-induced Olig2 overexpression and blocked IR-driven TUJ1 reduction in SCM. To further validate CA9’s role in IR-driven stem phenotype acquisition, sphere formation assays were performed. In our previous work [10], NS formed in suspension in SCM after 20–30 days. Here, we assessed early changes by monitoring adherent neurospherical structures as an initial NS formation indicator. In D1 and SRC3 cells transfected with shCTR, IR potentiated morphological cellular changes in SCM, leading to the formation of early adherent neurospherical structures at 72h post-IR. However, shCA9-transfected cells did not exhibit these changes in response to IR (Fig. 6C and Supplementary Figure S5). Finally, we investigated CA9’s role in early extracellular acidification linked to the mitochondrial metabolic shift induced by IR in cells undergoing dedifferentiation in SCM, in order to connect extracellular acidosis to IR-driven phenotypic plasticity. To assess this, OCR and ECAR were measured in SRC3 cells expressing shCTR or shCA9. Basal analysis and energy maps (Fig. 6D) showed that stable CA9 knockdown with shCA9-1 or shCA9-4 completely prevented the IR-induced ECAR increase at 48h post-IR in SCM, without affecting OCR increase. These results suggest that CA9 contributes to extracellular acidosis induced by IR during early dedifferentiation, with increased mitochondrial O_2_ consumption preceding the CA9-associated extracellular acidification.

## Discussion

Despite extensive preclinical and clinical research, no new pharmacologic or antibody-based therapies have significantly improved GBM prognosis since the TMZ/Radiotherapy (60 Gy total dose in 30 daily fractions of 2 Gy) combination in 2005 [2]. However, adding TTF to TMZ after chemoRT has recently shown improved overall (OS) and progression-free survival (PFS) in GBM patients [3]. In combination with chemoRT, several drugs, including Cilengitide (αvβ3/αvβ5-integrin inhibitor), Nivolumab (anti-PD-1 antibody), and Bevacizumab (BVZ, anti-VEGF antibody), failed to enhance OS in phase III trials for newly diagnosed GBM [61], though retrospective analysis suggests BVZ may benefit Proneural GBM patients [61]. The lack of effective treatments highlights the urgent need to understand resistance and adaptive mechanisms in these aggressive tumors. RT is a cornerstone of GBM treatment, but resistant clones, mainly GSC, can survive and be selected under RT-induced stress. This process is a well-known driver of tumor repopulation and the development of a more resistant secondary GBM [5]. Beyond clonal selection, we identified another mechanism, showing in vitro that subtoxic, clinically relevant IR doses trigger a survivin-mediated plasticity process, shifting resistant differentiated GBM cells towards a more stem-like state [10]. This phenotypic shift leads to a sustained increase in their self-renewal and tumorigenic potential, along with heightened expression of multiple stemness markers.

To investigate early dedifferentiation mechanisms induced by IR, we applied our previous methodology [10] to several primary GBM cell lines, focusing on short-term adaptive mechanisms during the first steps of the phenotypic shift (1 to 7 days post-IR). We analyzed early metabolic changes triggered by IR, known to alter energy production and biosynthesis in GBM cells and GSC [25, 26, 28]. This approach helps identify early targets enabling long-term phenotypic adaptation to metabolic stress from therapies like RT, which may drive tumorigenic rebound and recurrence. Additionally, evidence suggests that GBM cell differentiation status is linked to specific metabolic profiles, influencing tumorigenicity and radioresistance [32–34].

### Interplay between mitochondrial metabolism and glioblastoma cell stemness

In this study, we first characterized the basal metabolic state of several GSC cultures and their differentiated progenies. All GSC-enriched NS cell lines, despite marked molecular heterogeneity that can be seen through the presence of different molecular subtypes, exhibited higher OXPHOS-related O_2_ consumption and mitochondrial ATP production than differentiated cells. This increased OCR is associated with elevated extracellular acidification, which correlates in most of our GSC lines with CA9 expression, an enzyme that converts CO_2_ into H^+^  + HCO_3_^−^, as seen in breast and GBM CSC [57–59]. Other mechanisms of extracellular acidosis should be explored, as lactate/H^+^ extrusion or H^+^ export via Na^+^ /H^+^ exchangers (NHE) may also contribute to the ECAR increase in GSC. Indeed, MCT4 and NHE9, involved in stemness maintenance, are overexpressed in these cells [62, 63]. Consistent with previous studies, undifferentiated GBM cells favor OXPHOS, while differentiated cells rely almost exclusively on glycolysis [32, 34], similar to breast CSC [64]. This metabolic difference may arise from GSC oxidizing substrates like glutamine, fatty acids or extracellular lactates as observed in GBM cells and GSC in vitro and in vivo [28, 30, 65, 66], potentially enhancing mitochondrial-driven biosynthesis, OXPHOS and tumor repopulation. To support this, we analyzed the GSE54791 Dataset from GEO which contained RNA-seq data from three paired human GSC and GDC cultures [46] and observed a strong tendency to the overexpression of genes linked to Fatty Acid Oxidation (FAO) in GSC compared to their differentiated progeny, as wells as a similar trend for genes involved in glutamine-linked energetic metabolism. Of note, these RNA-seq data also confirmed our observations in GSC, with a significant increase in TCA and OXPHOS-related genes expression in GSC as well as an increase of glycolysis-related genes in paired GDC (Supplementary Figure S6).

### Glioblastoma dedifferentiation via CA9-driven early metabolic shift

We next demonstrated that IR triggers an early metabolic shift from glycolysis to OXPHOS during the initial stages of dedifferentiation in permissive stem medium. This reprogramming, marked by increased OCR and mitochondrial ATP production without lactate accumulation, was sustained over time. As glucose uptake remained unchanged in SCM 3Gy conditions, other substrates like fatty acids or glutamine may be fueling this shift, similar to glutaminolytic adaptations in GBM under mTOR-targeted therapy [65]. From 48h post-IR, OCR increase coincided with extracellular acidification and overexpression of GSC markers (Olig2, Sox2, Nestin, Survivin). We showed that IR induces an early rise in stem markers, initiating a long-term dedifferentiation process [10]. This increase, along with extracellular acidosis, occurred only in permissive stem medium post-IR, suggesting a link to dedifferentiation. An acidic environment is known to support GSC stemness [40] and enhance self-renewal [41]. To investigate the role of IR-induced acidosis in plasticity, we blocked extracellular acidification.

To identify the source of the ECAR increase, we analyzed extracellular H⁺ production. In tumor cells, lactate overproduction from glycolysis is typically the main driver of pHe reduction [35]. However, our model shows reduced aerobic glycolysis without an increase in extracellular lactate, ruling out MCT transporters in post-IR acidosis. Instead, pHe decrease may result from CO₂ overproduction [67, 68], possibly via enhanced Pentose Phosphate Pathway (PPP) and/or mitochondrial metabolism (TCA cycle and Pyruvate Dehydrogenase), consistent with increased OXPHOS-linked O₂ consumption. This CO₂ is converted into HCO_3_^−^ and H⁺ by membrane-bound CA9 and CA12, contributing to acidosis [35]. HCO_3_^−^ then re-enter the cell to buffer pHi, maintaining optimal functions at physiological pH. We and others have shown that CA9, like CA12, is overexpressed in GBM compared to normal brain tissue [56, 57], and that GSC often express higher CA9 than differentiated cells [59], similar to breast CSC [58]. Both CA9 and CA12 have been linked to poor prognosis in GBM patients [57]. CA9 expression is typically linked to hypoxia via the PI3K/Akt pathway but can also be regulated by the mitogen-activated protein kinase (MAPK) pathway, independently of hypoxia [69]. We showed that, unlike CA12, CA9 is specifically overexpressed by IR in stem medium during early and late dedifferentiation stages. This is among the first evidence that IR can induce CA9 expression also observed in rectal adenocarcinomas post-chemoRT [70]. Additionally, CA9 inhibition has been linked to radio- and chemosensitization in both GBM and colon cells [57, 71–73]. Its downregulation in our model confirmed its role in extracellular acidification and early stem-like features (Olig2 expression, neurospherical structure formation) post-IR. However, OCR increase and CA12 expression remained unchanged after CA9 knockdown, suggesting OCR rise post-IR precedes acidosis and CA12 cannot compensate for CA9 loss.

In summary, our results show that CA9 links IR-induced early metabolic reprogramming to dedifferentiation via extracellular acidification in GBM cells. Two key questions remain: (i) the mechanism behind IR-driven overactivation of the mitochondrial respiratory chain and (ii) the nature of the substrates or metabolites producing enough NADH/FADH2 for increased OXPHOS and CO_2_ generation post-IR. IR has been linked to enhanced OXPHOS activity and ROS production in GBM and other cancers [25, 28, 64, 74]. IR may also activate mitochondrial Complex II [75], a TCA component via its succinate dehydrogenase activity, generating superoxide [76]. Survivin, upregulated early and throughout IR-induced dedifferentiation, was shown to stabilizes Complex II, enhancing OXPHOS and ROS production in GBM cells [77]. It would be interesting to determine if IR-induced survivin drives OXPHOS activation via Complex II, mediating post-IR plasticity. Another key question is identifying the substrates sustaining OXPHOS and CO_2_ production, triggering acidosis-driven plasticity. FAO is linked to radioresistance in GBM cells and NSC [28, 78, 79], serving as pro-invasive and immune evasion mechanisms in GBM cells [80, 81]. Additionally, glutamine consumption can be activated in GBM cells under therapy [65, 82] or prolonged acidosis in tumor cells [83], potentially creating an auto-amplification loop in our model. Glutamine, explored as a strategy to sensitize cancers to treatment [84], also generate the TCA intermediate α-ketoglutarate, a key factor in maintaining embryonic stem cell pluripotency [85]. Moreover, in glycolysis-impaired cells, glutaminolysis enhances oxidative PPP activity, increasing CO_2_ production and extracellular acidification [67]. Understanding the roles of FAO, glutaminolysis, and PPP in IR-induced metabolic adaptation will improve insights into IR-mediated dedifferentiation in GBM.

### Study considerations

This study has several limitations that will need to be addressed in our future work in order to increase its impact and confirm the CA9 target at the clinical level. First, it is necessary to confirm at the preclinical level that targeting CA9 could block the IR-induced metabolic shift and dedifferentiation of GB cells leading to faster recurrence. To this end, we plan to conduct experiments on 3D cerebroid models implanted with primary GSC lines [86]. We also plan to perform orthotopic xenografts of irradiated GB cells from patients in nude mouse models, as we routinely do in the laboratory [10, 45], with and without CA9 inhibitors, some of which are already in clinical use [87]. Next, although we reproduced our results in 7 primary GB cell lines from 7 different patients, and although the molecular subtypes are quite heterogeneous among these cell lines (Supplementary Table 3), it must nevertheless be considered that this process of metabolic and phenotypic plasticity induced by IR may vary depending on certain types of GB. Particular attention should be paid to this molecular heterogeneity of GB, and the genetic and epigenetic characterization of the GB lines used needs to be correlated with the analysis of this radio-induced plasticity. Finally, from a more mechanistic point of view, as mentioned earlier in the discussion, it is essential to identify the nature of the substrates (glutamine, fatty acids, etc.) that fuel the post-IR increase in OXPHOS and the subsequent dedifferentiation process, in the absence of a proven increase in glucose utilization. Several studies have shown that, unlike GDC, GSC can use these alternative substrates [29, 30], and we have also been able to confirm, through published RNA-seq data analysis of three paired GSC and GDC lines (GSE54791, [46]) that many genes linked to FAO pathway are overexpressed in GSC (Supplementary Figure S6). Our next investigations will therefore focus on determining these metabolic pathways, particularly FAO, during the process of IR-induced dedifferentiation and, if possible, identifying a key player among these pathways whose inhibition could constitute a new therapeutic approach in the radiosensitization of GB.

## Conclusions

From a clinical standpoint, this study highlights the therapeutic potential of targeting molecular mediators, like CA9 and survivin, involved in the early adaptive response to IR. Inhibiting these pathways may prevent long-term phenotypic shifts toward a stem-like state, hinder GSC repopulation, reduce tumor recurrence and ultimately enhance RT effectiveness in GBM patients.

## Supplementary Information

Below is the link to the electronic supplementary material.

Supplementary Material 1.

Supplementary Material 2.

## Data Availability

Not applicable.

## Abbreviations

BVZ: Bevacizumab
CA: Carbonic anhydrases
CA9: Carbonic anhydrase IX
CA12: Carbonic anhydrase XII
CMV: Cytomegalovirus
CSC: Cancer stem cell
ECAR: Extracellular acidification rate
FAO: Fatty acid oxidation
FCS: Fetal Calf Serum
GBM: Glioblastoma
GDC: Glioblastoma differentiated cell
GSC: Glioblastoma stem cell
IR: Ionizing radiation
MCT: Monocarboxylate transporter
NHE: Na+/H + exchanger
NS: Neurosphere
OCR: Oxygen consumption rate
OS: Overall survival
OXPHOS: Oxidative phosphorylation
PFS: Progression free survival
PPP: Pentose phosphate pathway
ROS: Reactive oxygen species
RT: TMZ-Radiotherapy
SCM: Stem cell medium
SCTF: Stem cell transcription factor
TCA: Tricarboxylic acid cycle
TMZ: Temozolomide
TTF: Tumor treating fields
WHO: World Health Organization
