# Supplementary material for: Early metabolic reprogramming and carbonic anhydrase IX-mediated extracellular acidification drive radiotherapy-induced glioblastoma cell dedifferentiation
Source: Acta Neuropathol Commun. 2025 Nov 28;14:1. doi: 10.1186/s40478-025-02161-2 (PMC12764069; doi:10.1186/s40478-025-02161-2)

Supplementary Data

1. Supplementary Methods

1.1 GSC isolation from GBM patient samples and cell culture

All experiments using the seven GSC-enriched neurosphere (NS) cell lines were realized between the 2nd and 12th passages in order to avoid any stem cell characteristic loss. In detail, GBM differentiated cells (GDC) were plated (7.5 × 10^3 cells/cm^2) one day before IR (subtoxic dose of 3 Gy). One day later, irradiated cells or not (control, CTR) were either kept in culture in FCS medium (negative control) or in SCM, a stem-permissive medium without FCS but enriched in EGF/bFGF (Peprotech) and B27/N2 supplements (Life Technologies). Experiment parameters were measured at 24h, 48h, 72h, 120h or one week post-IR. For some GSC cell lines, plated cells were observed by microscopy (Nikon Diaphot) under a 10X objective and the forming 3D neurospherical structures were counted 72h post-IR for each condition. In some experiments, the long-term dedifferentiation process was applied as described in [10] for at least 4 weeks.

1.2 Oxygen consumption rate and extracellular acidification rate (Seahorse XF assay)

The day before the assay, initial GSC (NS) and irradiated or CTR GDC cells were both plated in 24/96-well microplates (Agilent) on laminin (1.5 µg/cm^2, Sigma-Aldrich) at approximately 90,000 cells per well (4 replicates per condition at least). Prior to this XF assay, cells were incubated at 37°C 0% CO_2 for 60min in assay medium (XF Base Medium, Agilent) supplemented by Glutamine 2mM (Life technologies), Glucose 10mM (Sigma-Aldrich) and Sodium Pyruvate 2mM (Sigma-Aldrich) as finals concentrations and adjusted to pH 7.4. Using MitoStress kit (Agilent), we measured basal OCR, basal ECAR and Maximal Respiratory parameters. Normalization was performed by determining the protein content in each well as previously described [27].

1.3 ATP measurement

96-well plates were seeded with 10,000 cells per well in 80µL of DMEM-F12 cell culture medium (Lonza). 20µL of inhibitors (Sigma-Aldrich), oligomycin, antimycin, sodium iodoacetate or a mix of sodium iodoacetate and oligomycin or antimycin were added or not at final concentration of 15µM, 5µM and 100µM, respectively. After 1hr at 37°C CO2 0.5%, 100µL of Cell Titer-Glo were added to each well to a final volume of 200µL. After 10min at RT with and then without agitation, the plate was read by luminometer (FluoStar, Optima). By comparing the different conditions, global ATP and percentages of both glycolytic and mitochondrial ATP were determined [27].

1.4 Quantitative real-time RT-PCR

Total RNA was isolated either from primary NS, FCS-differentiated cells or dedifferentiating cells using RNeasy kit (QIAGEN) and then reverse-transcribed using iScript cDNA synthesis kit (Bio-Rad) according

to manufacturer's instructions. Quantitative RT-PCR was performed with a StepOnePlus PCR detection system (Applied Biosystems) using iQ SYBR Green Supermix (Bio-Rad). β2-microglobulin (β2M) was used as endogenous control in the ΔCt analysis. The different primers (Eurogentec) used in this study are CA9, (Forward: 5'-TTTGCCAGAGTTGACGAGGC-3'; Reverse:5'-GCTCATAGGCACTGTTTTCTTCC-3'), CA12 (Forward:5-AGTGAACGGTTCCAAGTGGAC-3'; Reverse: 5'-CCACACGACGGGTACTTCT-3') or were described in [10] for Olig2 and β2M. Concerning the comparison analysis for CA9 expression between primary GBM patient samples (GSC and their differentiated counterparts), conventional GBM cell lines and normal brain samples (detailed in *Supplementary Table S2*), real-time qPCR reactions were carried out using the Fluidigm 96.96 dynamic array integrated fluidic circuits and the Biomark HD System (Fluidigm) according Advanced Development Protocol n° 37 (Toulouse GeT Platform). β2M was also used as endogenous control in the ΔCt analysis.

1.5 Western blotting

Cells were lysed in RIPA buffer complemented with cocktails of protease and phosphatase inhibitors (Sigma-Aldrich). 25µg of proteins were then separated on a 10% or 12.5% SDS-PAGE, electroblotted onto PVDF membranes (Amersham), which were blocked with 10% milk. The primary antibodies used here are the monoclonal rabbit antibody to CA9 (1:1000, Abcam Ab108351), the polyclonal goat antibody to CA12 (0.1 µg/mL, R&D, AF2190) or listed in [10].

1.6 Flow cytometry analyses

Direct immunofluorescence assay was performed by FACS as described [10]. For immunostaining of CA9 protein, the APC-conjugated antibody toward CA9 (1:20, R&D, FAB2188A) or the corresponding isotype control (1:20, R&D, IC003A) was used. To evaluate the marker expression, we determined the specific fluorescence index (SFI) using the mean fluorescence intensity (MFI). The SFI was calculated as previously described, with the following formula SFI = (MFI antibody−MFI isotype)/MFI isotype.

1.7 Neurospheres generation assay

For limiting dilution assay, as previously described [44], NS from GSC were dissociated and viable GSC were plated in 96 well-plates (16 wells/condition) at different cellular densities (1 to 50 cells/well) in order to assess their ability to generate secondary NS through limiting dilution assays. After 15 days, NS were counted by microscopy in each well.

2. Supplementary Tables

Supplementary Table S1. CA9 shRNA sequences.

Name	Supplier	Target	Selection Gene	Sequences
**ShCTR**	Qiagen	Unspecific	Neomycin	GGAATCTCATTCGATGCATAC
**Sh1 CA9**	Qiagen	CAIX	Neomycin	TTCAGCCGCTACTTCCAATAT
**Sh4 CA9**	Qiagen	CAIX	Neomycin	GCTGTCTCGCTTGGAAGAAAT

Supplementary Table S2. Commercial RNA samples from normal human brain.

Cell Types	Samples	Suppliers	Reference
**Neural Stem or Progenitor cells (NSC/NPC)**	Human Neural prenatal Progenitor Total RNA (Single Donor)	DvBiologics	pN003-r
	Human Astrocytes brain stem Total RNA (Single Donor)	ScienceCell	1845
	Human Adult Brain Stem Total RNA (Single Donor)	Agilent	R540053
**Adult Brain Cortex**	Human Brain Cerebral Cortex Total RNA (Single Donor)	Biochain	R1234042-10
	Human Cerebral Cortex Total RNA (Pool of 5 Donors)	Clontech	636561
	Human Frontal Cortex Total RNA (Pool of 4 Donors)	Clontech	636563
	Human Temporal Cortex Total RNA (Pool of 8 Donors)	Clontech	636564
	Human Parietal Cortex Total RNA (Pool of 4 Donors)	Clontech	636571
	Human Occipital Cortex Total RNA (Pool of 5 Donors)	Biochain	R1234062-P
	Human Frontal Cerebral Cortex Total RNA (Single Donor)	Agilent	540117
	Human Parietal Cerebral Cortex Total RNA (Single Donor)	Agilent	540143
	Human Occipital Cerebral Cortex Total RNA (Single Donor)	Agilent	540137
**Adult Brain White Matter**	Human White Cerebral Matter Total RNA (Single Donor)	Origene	CR562760

Supplementary Table S3. GSC molecular subtypes. *nd: not determined*

	Predicted Molecular Subtype (GLIOVIS)	
**Patients**	**Tumor bulk biopsy**	**Primary GSC line**
**C1**	*nd*	Classical
**D1**	*nd*	Classical
**G**	*nd*	*nd*
**I**	*nd*	Classical
**SRA5**	Proneural	Mesenchymal
**SRB1**	Mesenchymal	Proneural
**SRC3**	Classical	Classical

3. Supplementary Figures

Supplementary Figure S1

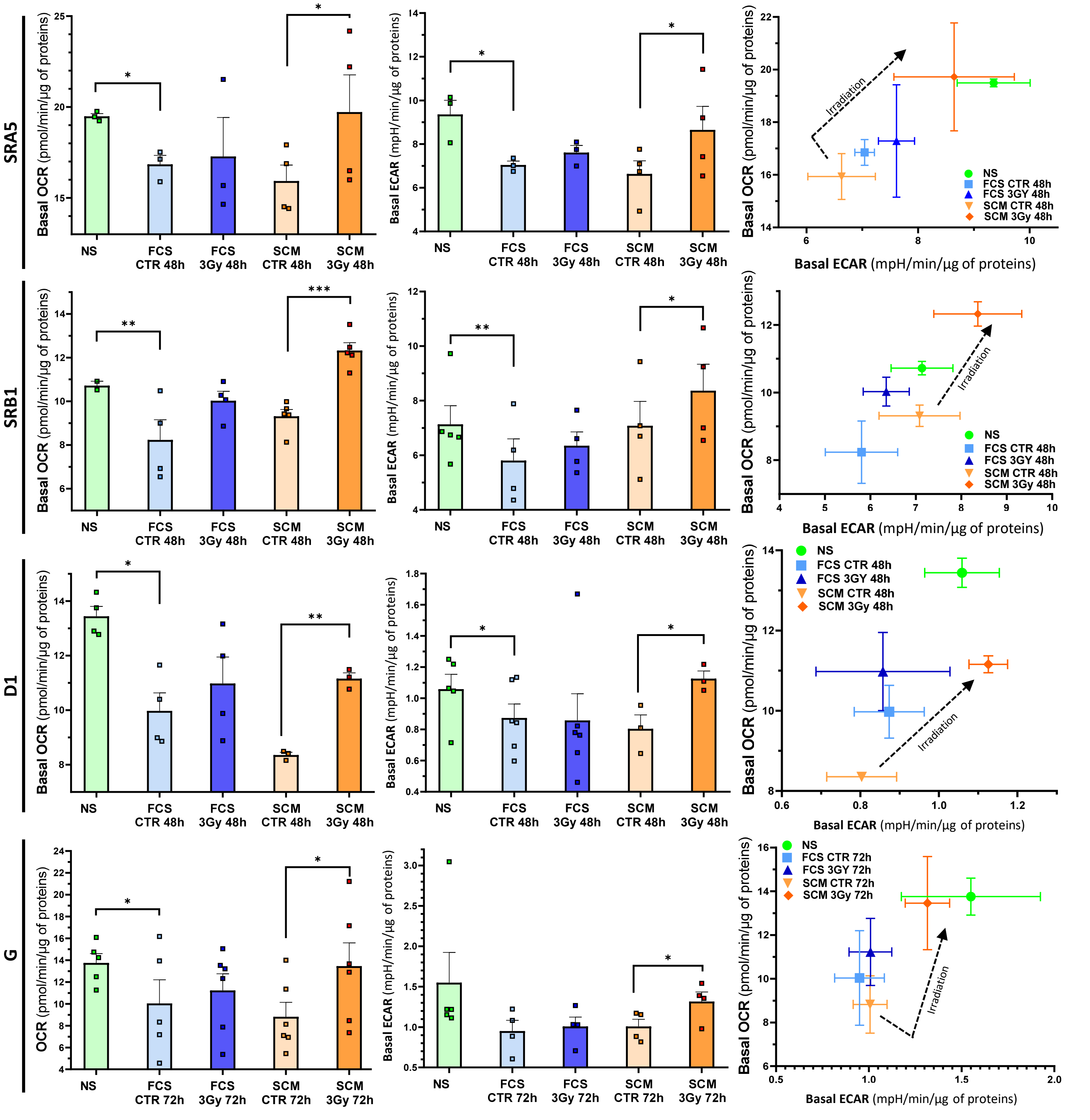

Supplementary figure S1: Early energetic metabolic shift during IR-induced dedifferentiation Differentiated GBM cells from SRA5, SRB1, D1 and G primary cell lines treated or not by a 3-Gy irradiation and placed 1 day after in either FCS or SCM medium for short-term culture (48h or 72h post-IR as indicated) were analyzed by Seahorse analysis for basal Oxygen Consumption Rate (OCR, pmol/min) and Extracellular Acidification Rate (ECAR, mpH/min) after normalization to total protein content. NS conditions were shown as controls for the stem condition. Results obtained in either FCS or SCM medium at 48h or 72h post-IR are shown as means±S.E.M. of at least three independent experiments. $^{*}P<0.05$, $^{**}P<0.01$, $^{***}P<0.001$. The corresponding energy phenograms were shown to illustrate the metabolic shift occurring at 48h or 72h post-IR in cells irradiated or not in either FCS or SCM medium.

Supplementary Figure S2

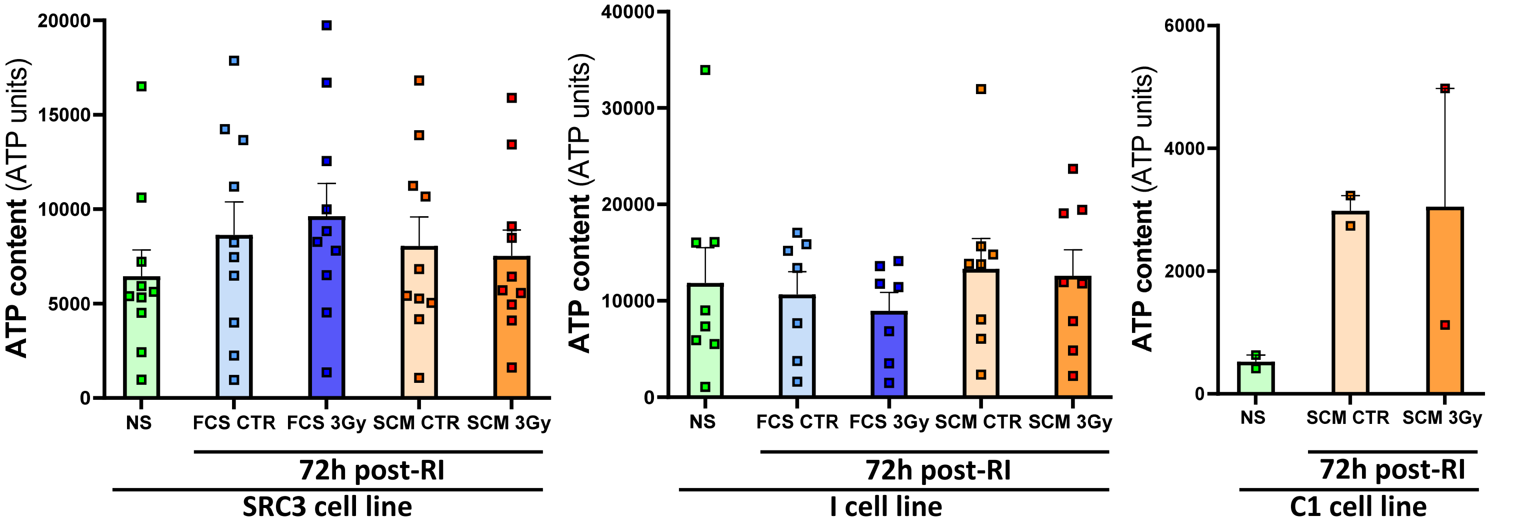

Supplementary figure S2: Total ATP content at the early stages of the dedifferentiation process. Differentiated GBM cells from the indicated patient cell lines treated or not by a 3-Gy irradiation and placed 1 day after in either FCS or SCM medium for short-term culture (72h post-IR) were analyzed for their total intracellular ATP content. Shown are the means±S.E.M. of at least 7 independent experiments, except for C1 cell line (n=2). NS parental cell results were shown as controls for the stem condition in each cell line.

Supplementary Figure S3

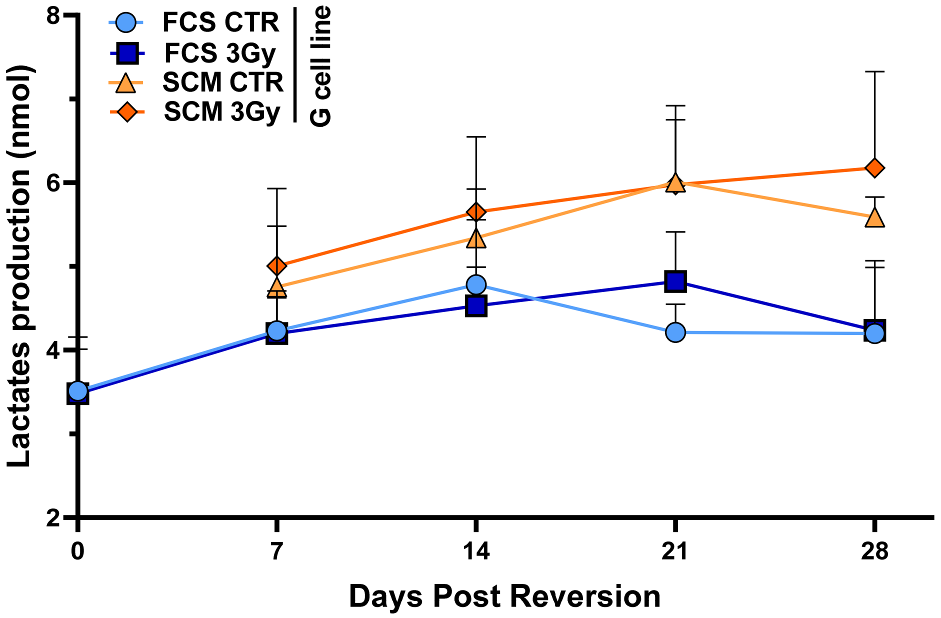

Supplementary figure S3: Lactate production all along the long term IR-induced dedifferentiation process. Differentiated GBM cells (G cell line) treated or not by a 3-Gy irradiation and placed 2 days after in either FCS or SCM medium for long-term culture were analyzed for lactate production in cell supernatants all along the dedifferentiation protocol at the indicated time points. Shown are the means±S.E.M. of four independent experiments.

Supplementary Figure S4

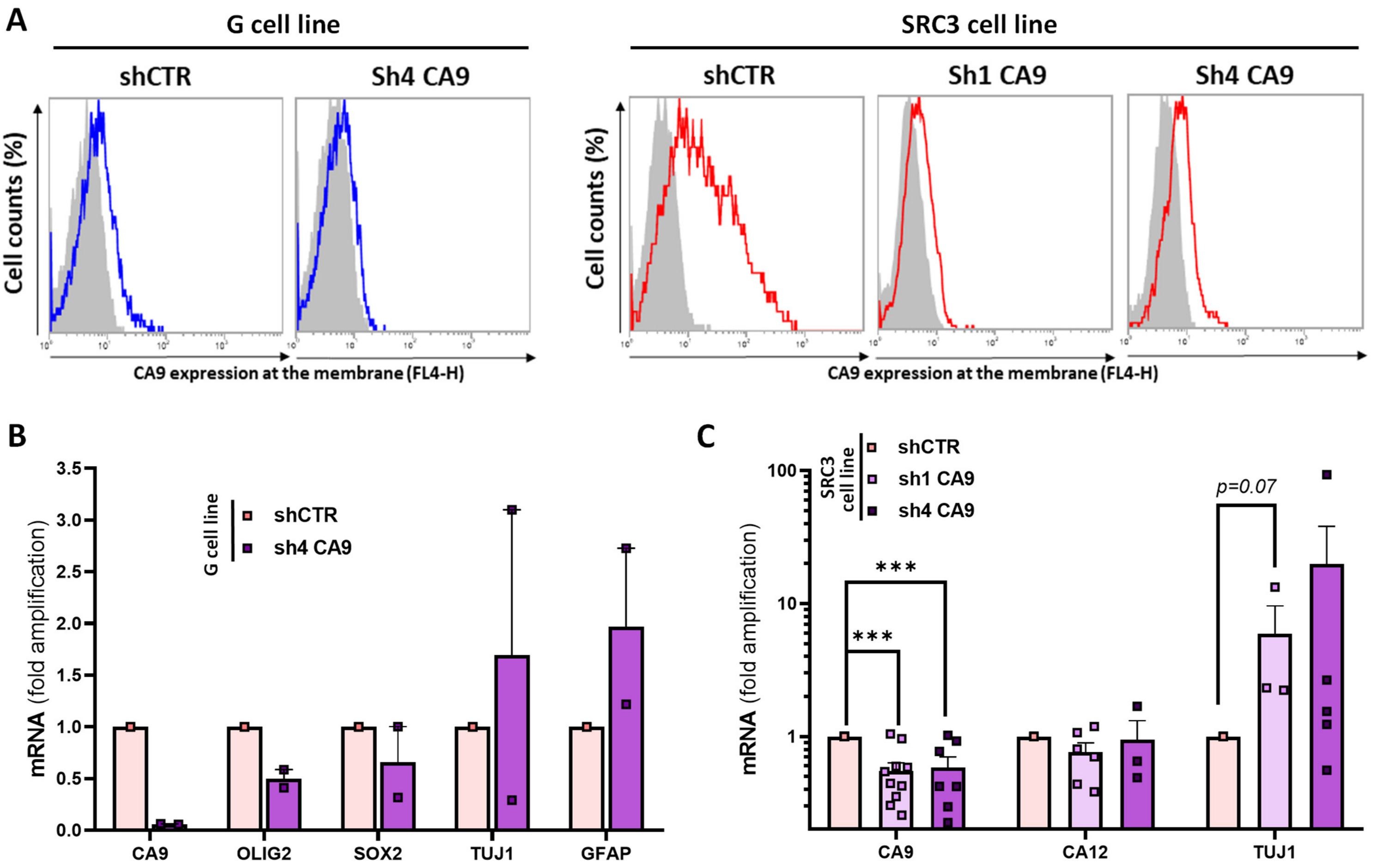

Supplementary figure S4: shRNA treatment efficacy on targeting CA9 and its consequences on stem and differentiation markers expression. Stably transfected SRC3 and G primary cell lines with shCTR or sh1/4-CA9 were harvested in order to check CA9 inhibition by flow cytometry **(A)** and its impact on several molecular factors expression by real-time qPCR **(B,C)**. **(A)** Immunofluorescence analysis performed by FACS of cell membrane CA9 in G (left panel, n=2) and SRC3 (right panel, n=1) cell lines. Representative FACS plot overlays using anti-CA9 antibody (in blue or red) and isotypic control (in grey) were depicted for each condition. **(B,C)** mRNA expression levels of CA9, Olig2, Sox2, TUJ1, GFAP in G **(B)** and SRC3 **(C)** primary cell lines. Shown are fold amplifications relative to the shCTR condition of two independent experiments for G cells and at least 3 independent experiments for SRC3 cells. ∗∗∗P<0.001.

Supplementary Figure S5

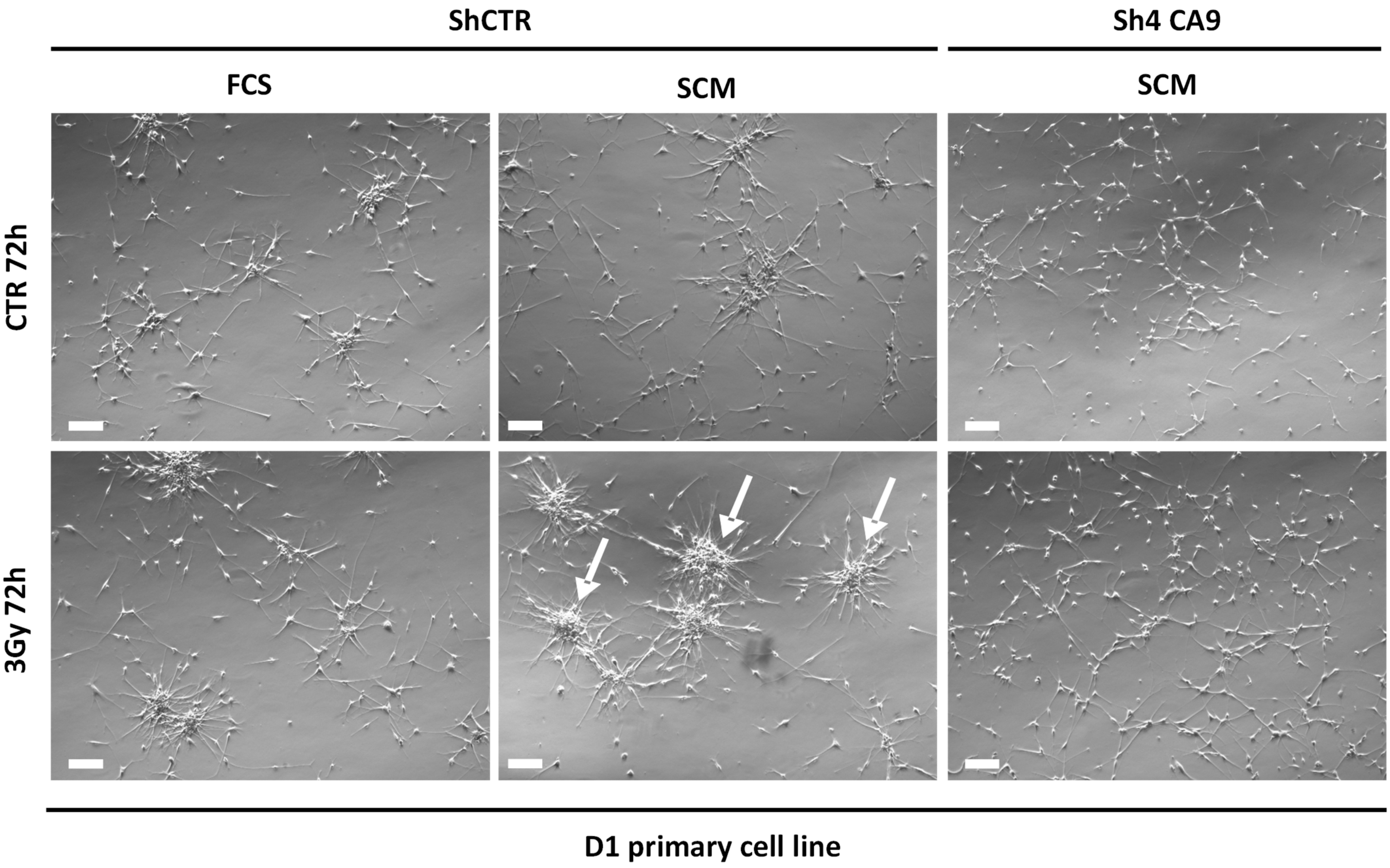

Supplementary figure S5: CA9-targeting shRNA treatment on neurosphere forming capacity post-IR. Phase-contrast photomicrographs of D1 cells stably expressing shCTR or sh4-CA9 subjected to the dedifferentiation protocol in SCM medium at 72h post-IR (Original magnification: × 10, scale bar: 6 µm, n=1). Arrows indicate the presence of neurospherical structures. Photomicrographs are representative of 3 different snapshots taken on 3 separate fields per condition.

Supplementary Figure S6 (panels A-C)

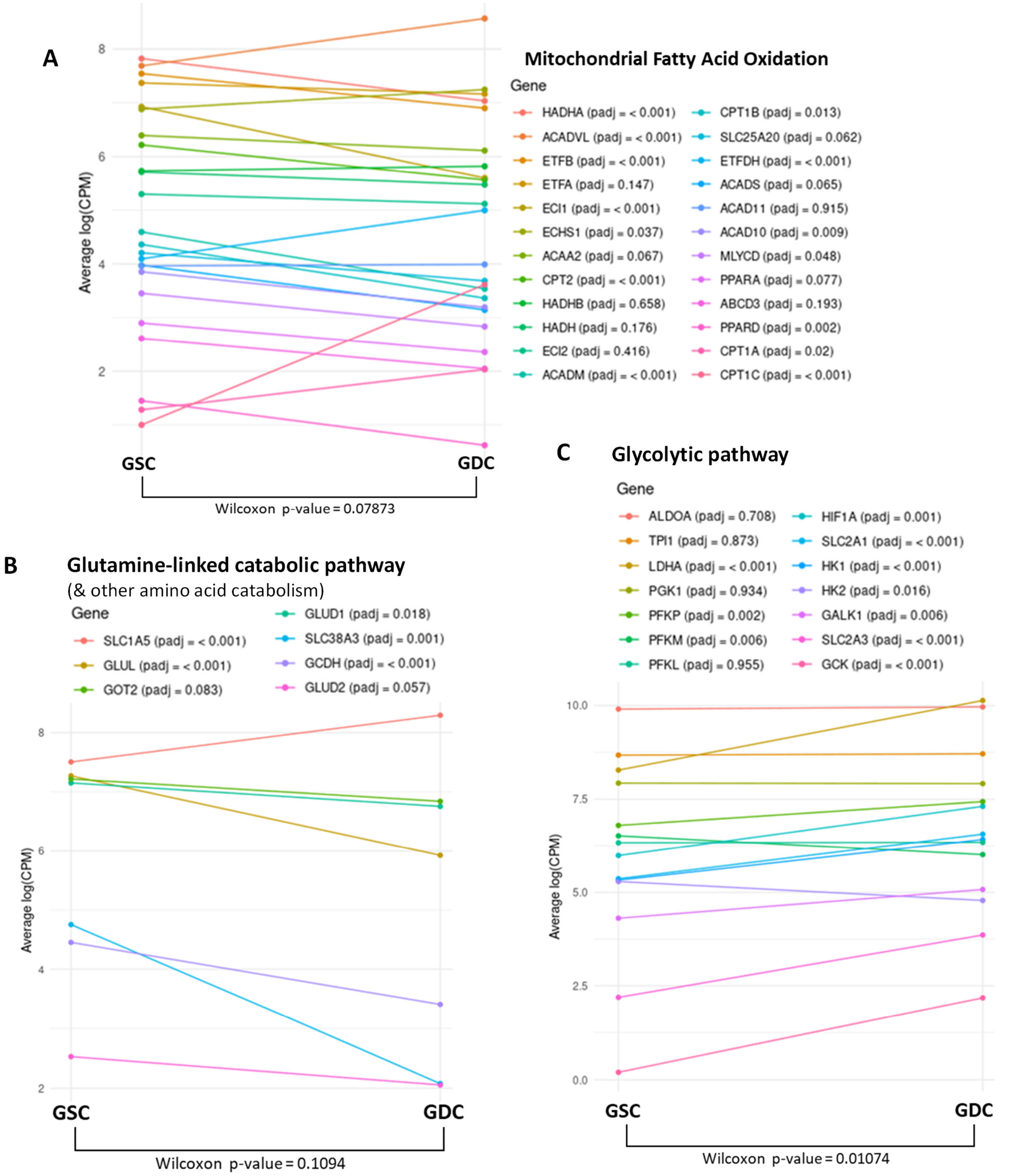

Supplementary figure S6: Differential expression analysis of metabolic genes between GSC and paired GDC. RNA-seq data were downloaded from GEO (GSE54791) comparing three different human GSC cell lines (MGG4, MGG6 and MGG8) and their matched differentiated progeny (GDC). Levels of expression corresponds to the average normalized expression (log2(CPM+1)) of the 3 cell lines and 3 replicates. Padj of each gene corresponds to the result of the differential analysis of GSC vs. GDC with limma. Paired samples Wilcoxon tests and associated p-values compare for each gene group the GSC and GDC conditions.

Supplementary Figure S6 (panels E-H)

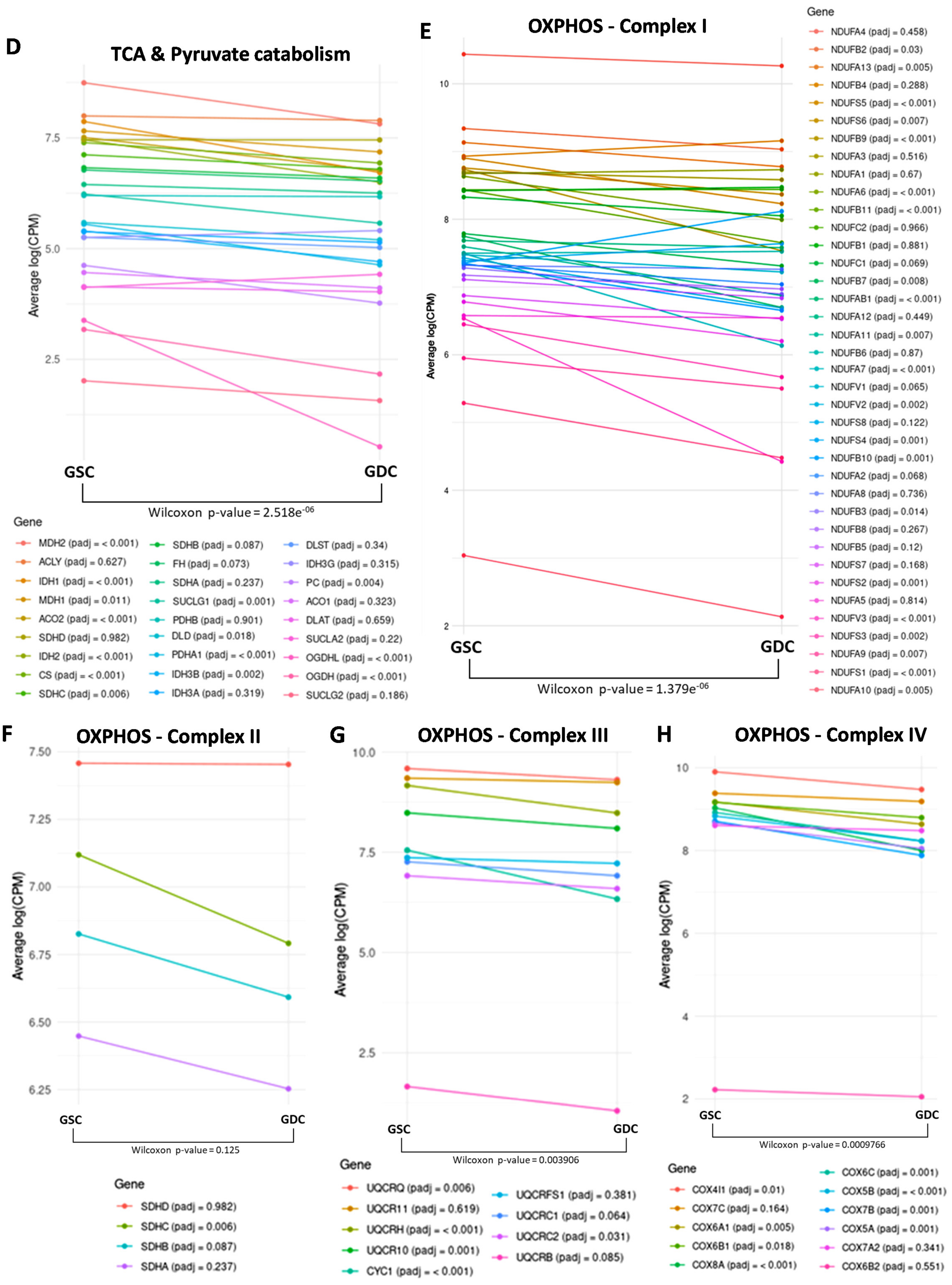

Supplement: Supplementary file 1 — Supplementary Material 1. [file 40478_2025_2161_MOESM1_ESM.pdf]
