# Supplementary material for: Early metabolic reprogramming and carbonic anhydrase IX-mediated extracellular acidification drive radiotherapy-induced glioblastoma cell dedifferentiation
Source: Acta Neuropathol Commun. 2025 Nov 28;14:1. doi: 10.1186/s40478-025-02161-2 (PMC12764069; doi:10.1186/s40478-025-02161-2)

Supplementary File - Scans

Figure 1 - C1 GSC
48h post-IR

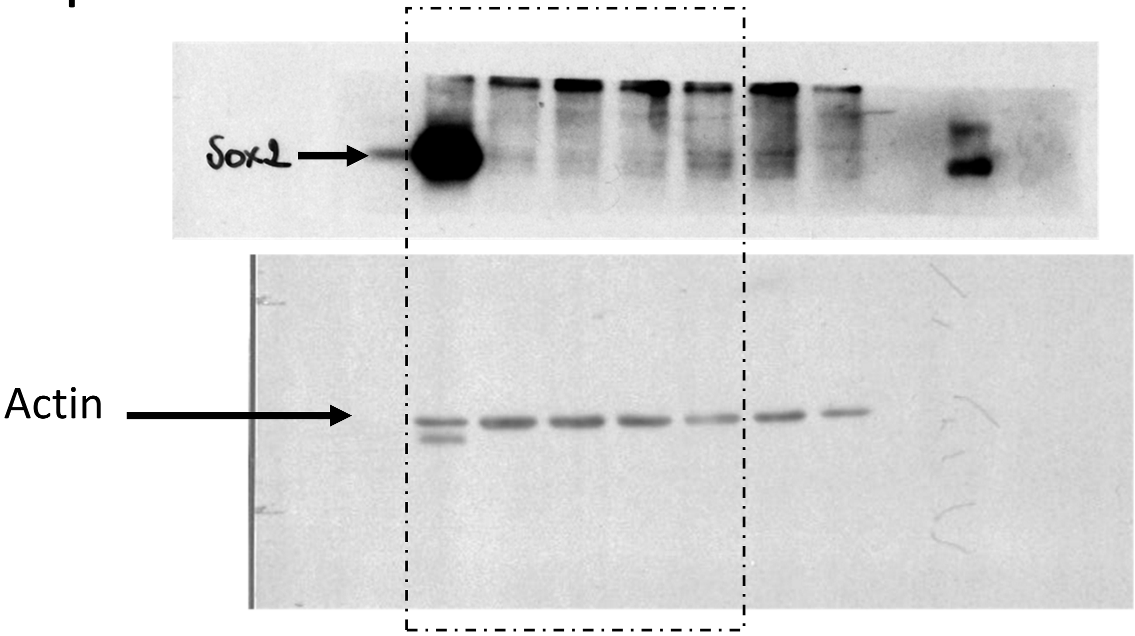

1 week post-IR

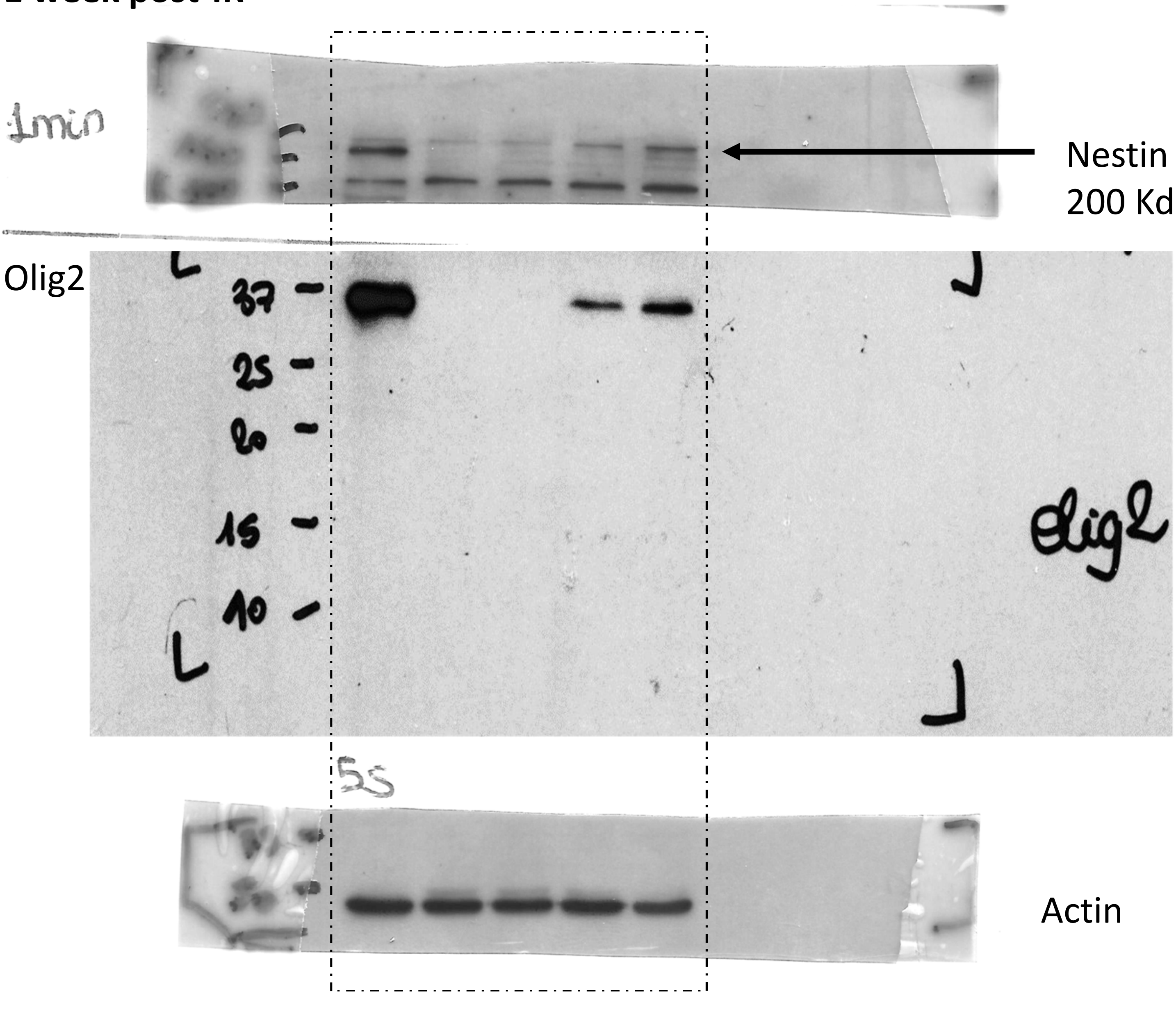

Figure 1 - G GSC 48h post-IR

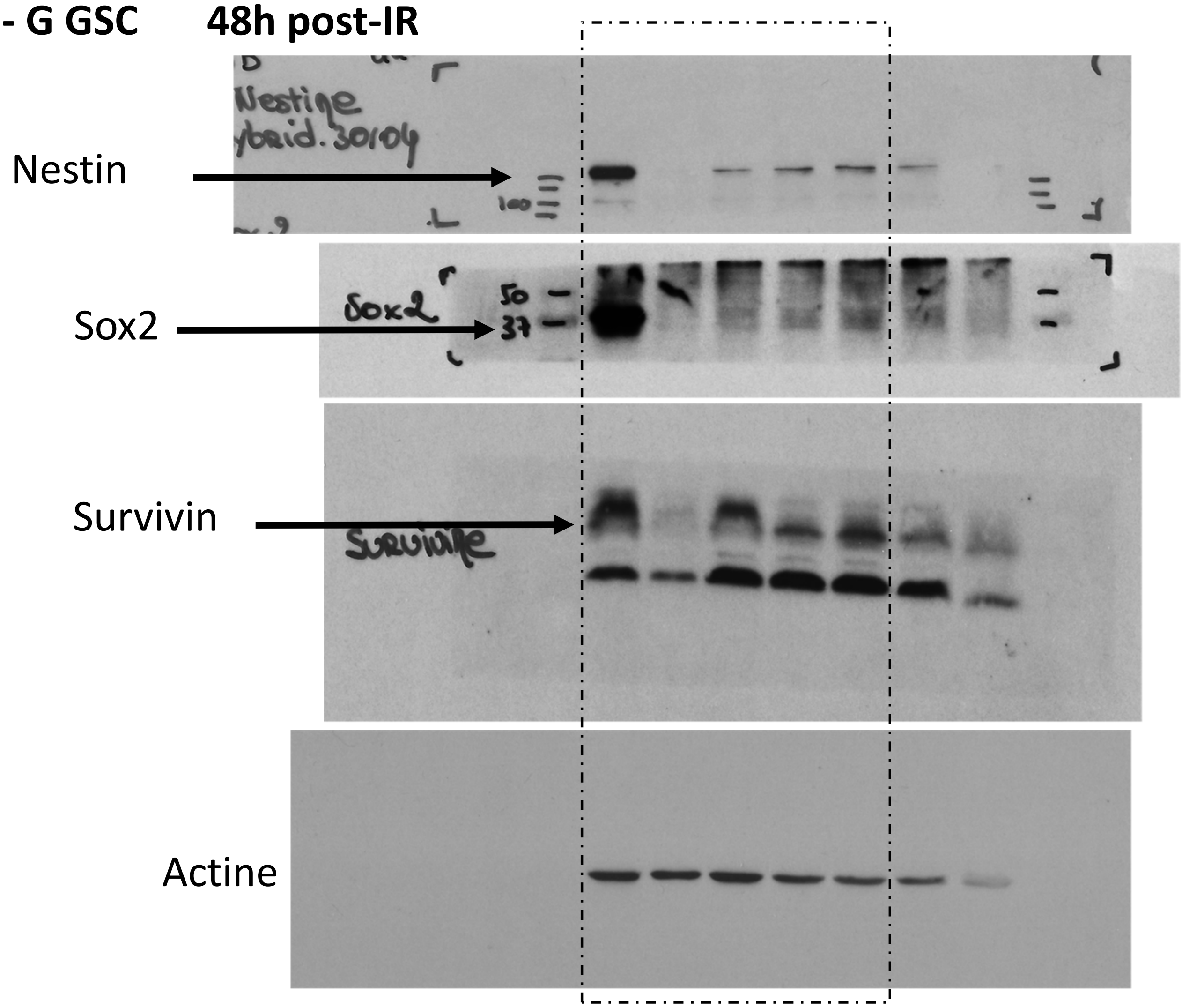

1 week post-IR

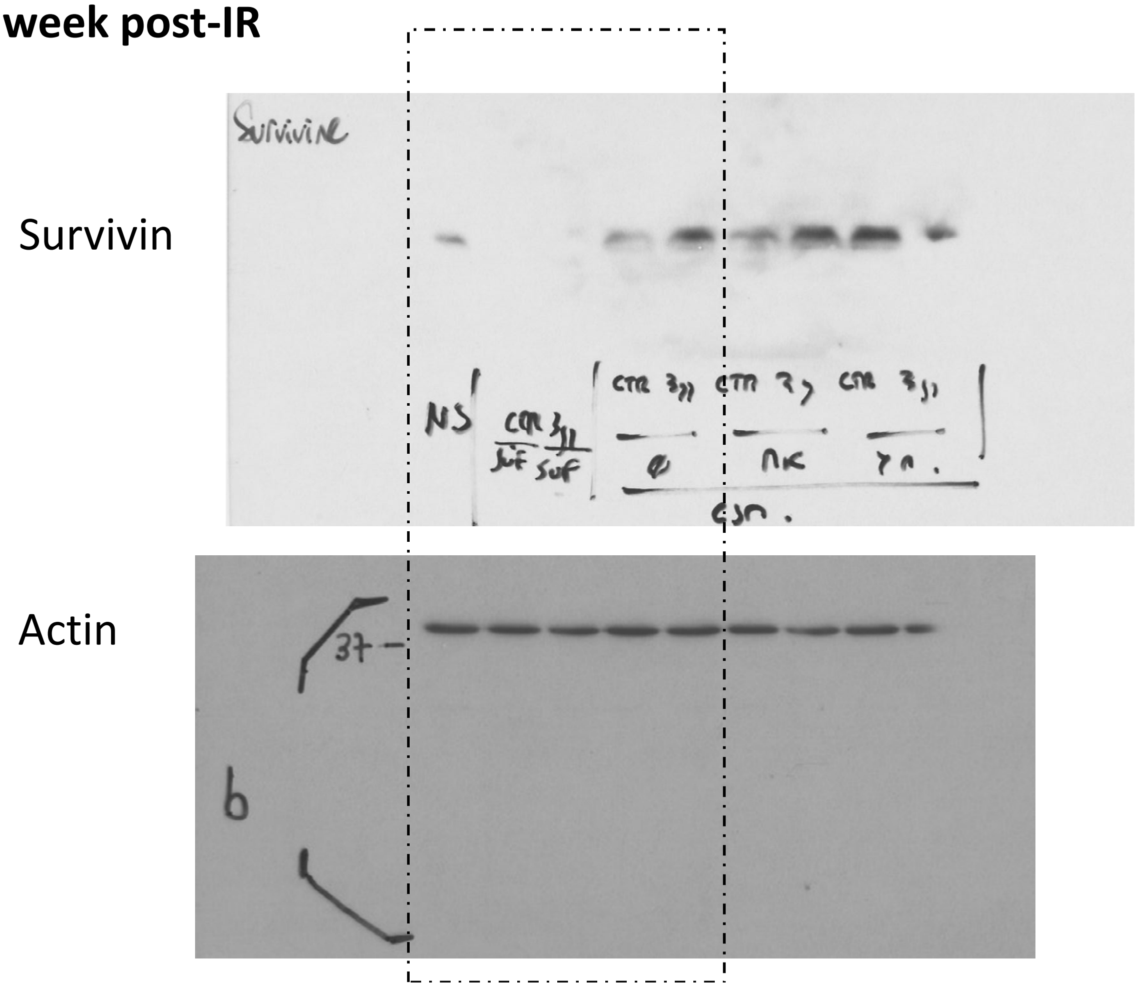

Figure 1 - SRA5 GSC

48h post-IR

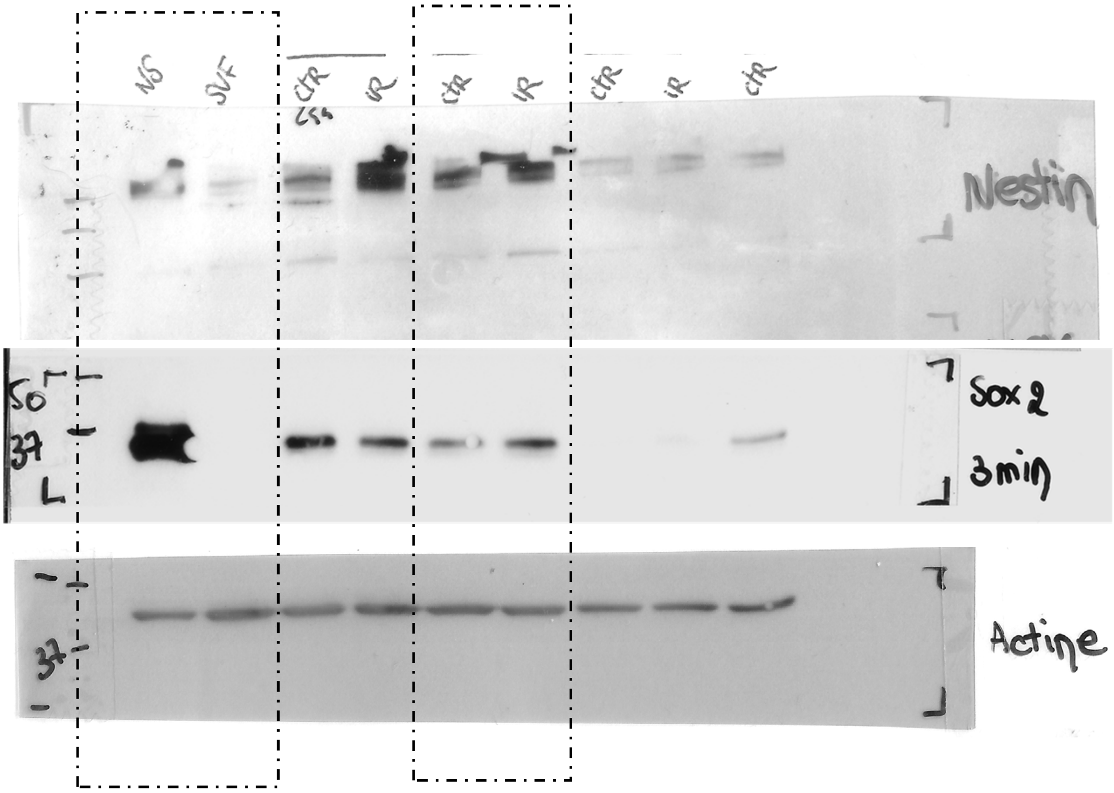

72h post-IR

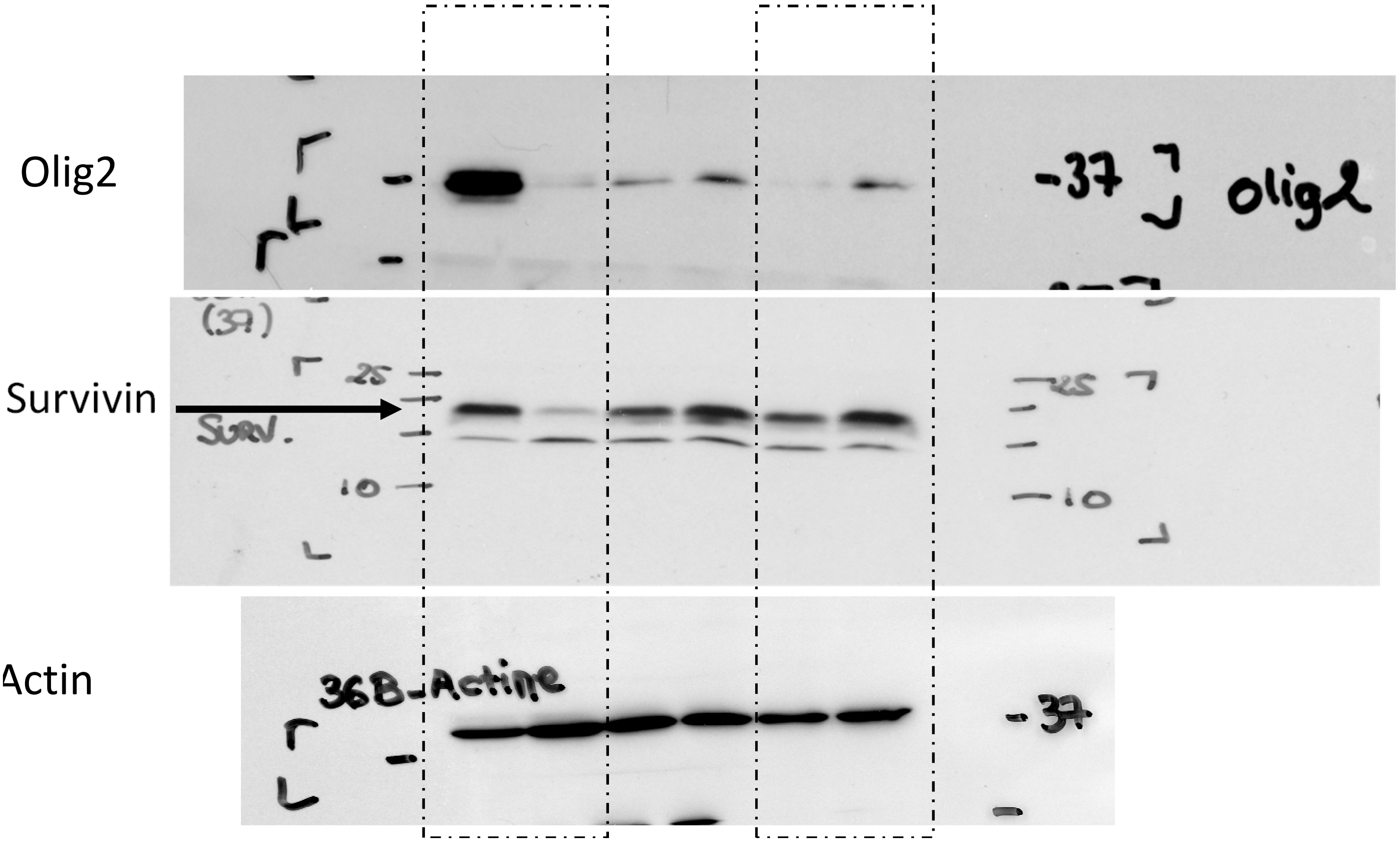

Figure 1 - I GSC

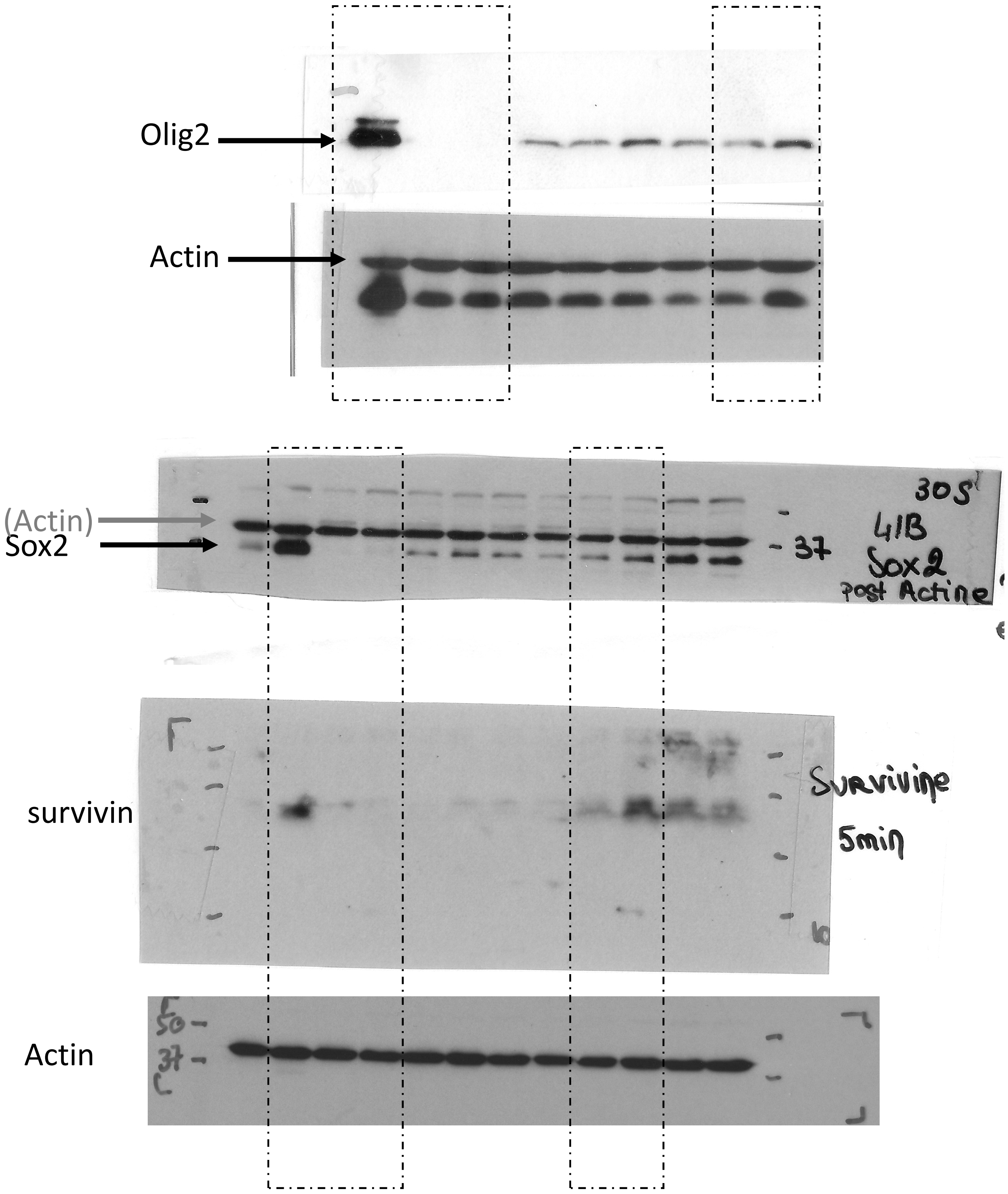

Figure 4E - I primary cell line

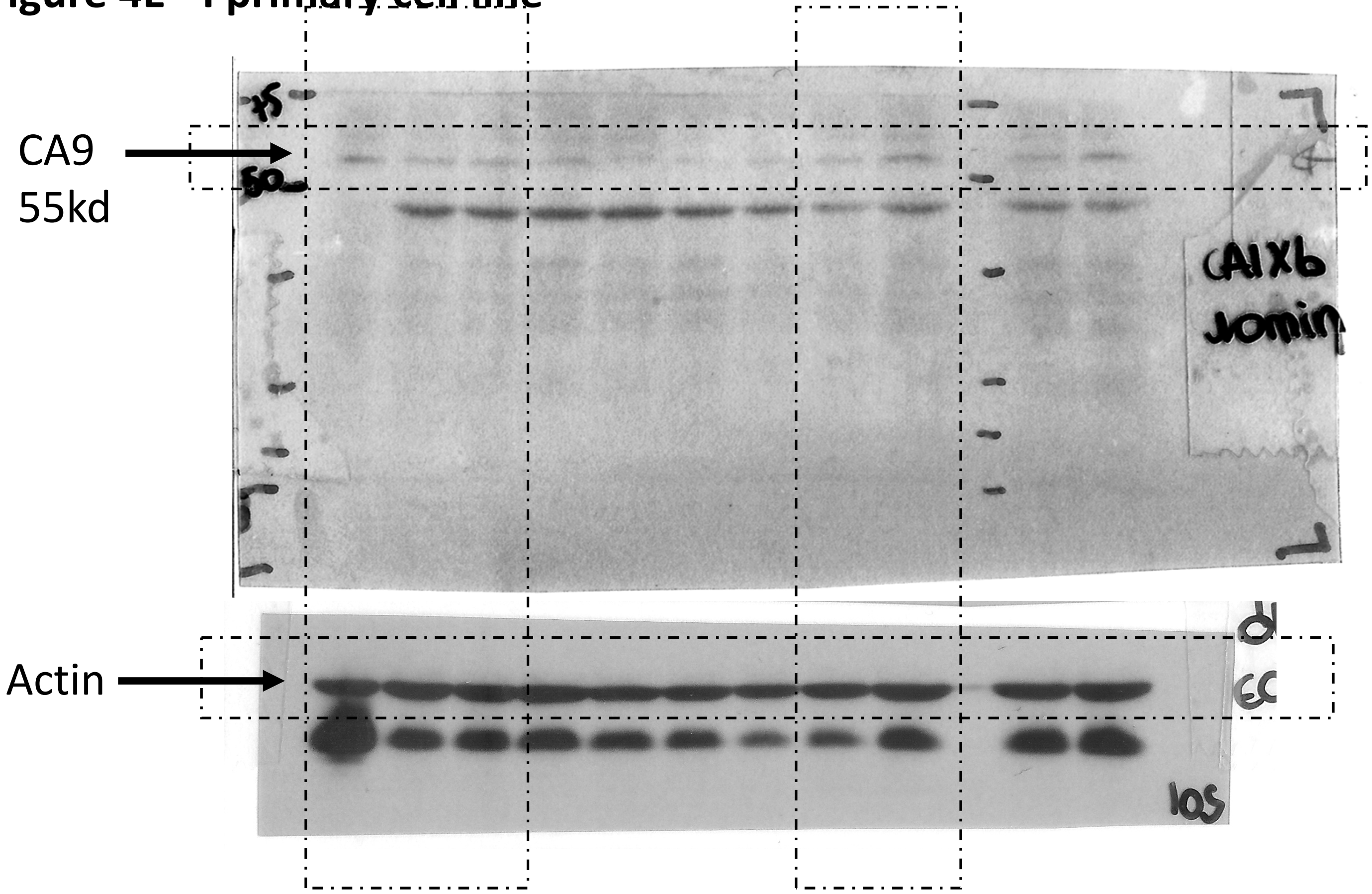

Figure 4E - G primary cell line

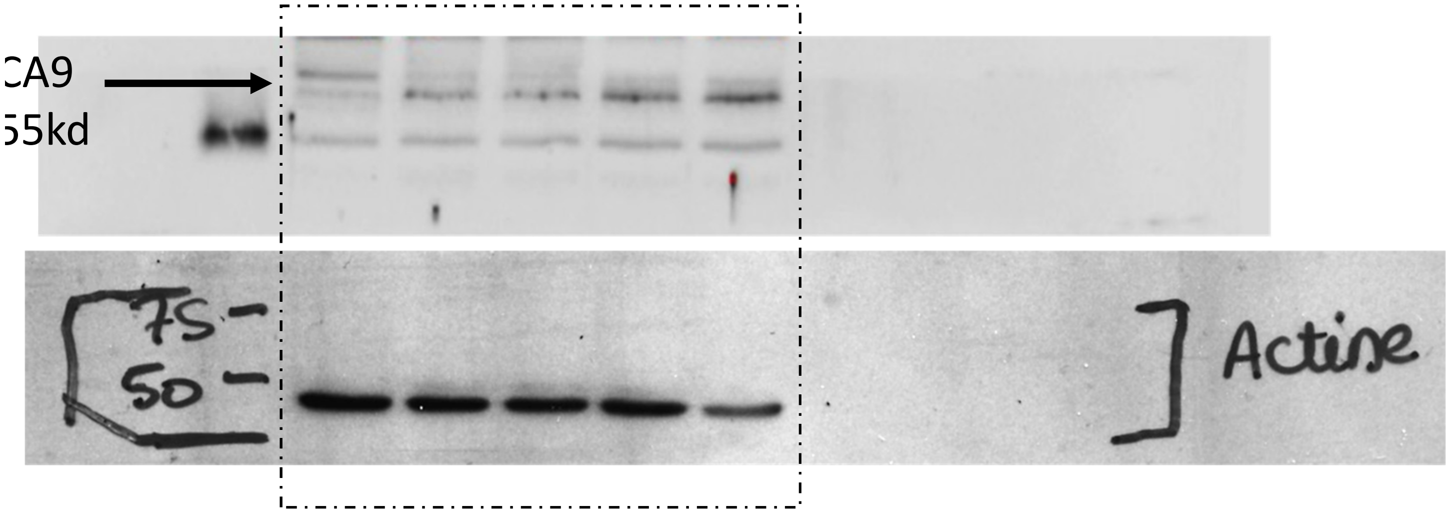

Figure 4G - long term dedifferentiation (I primary cell line)

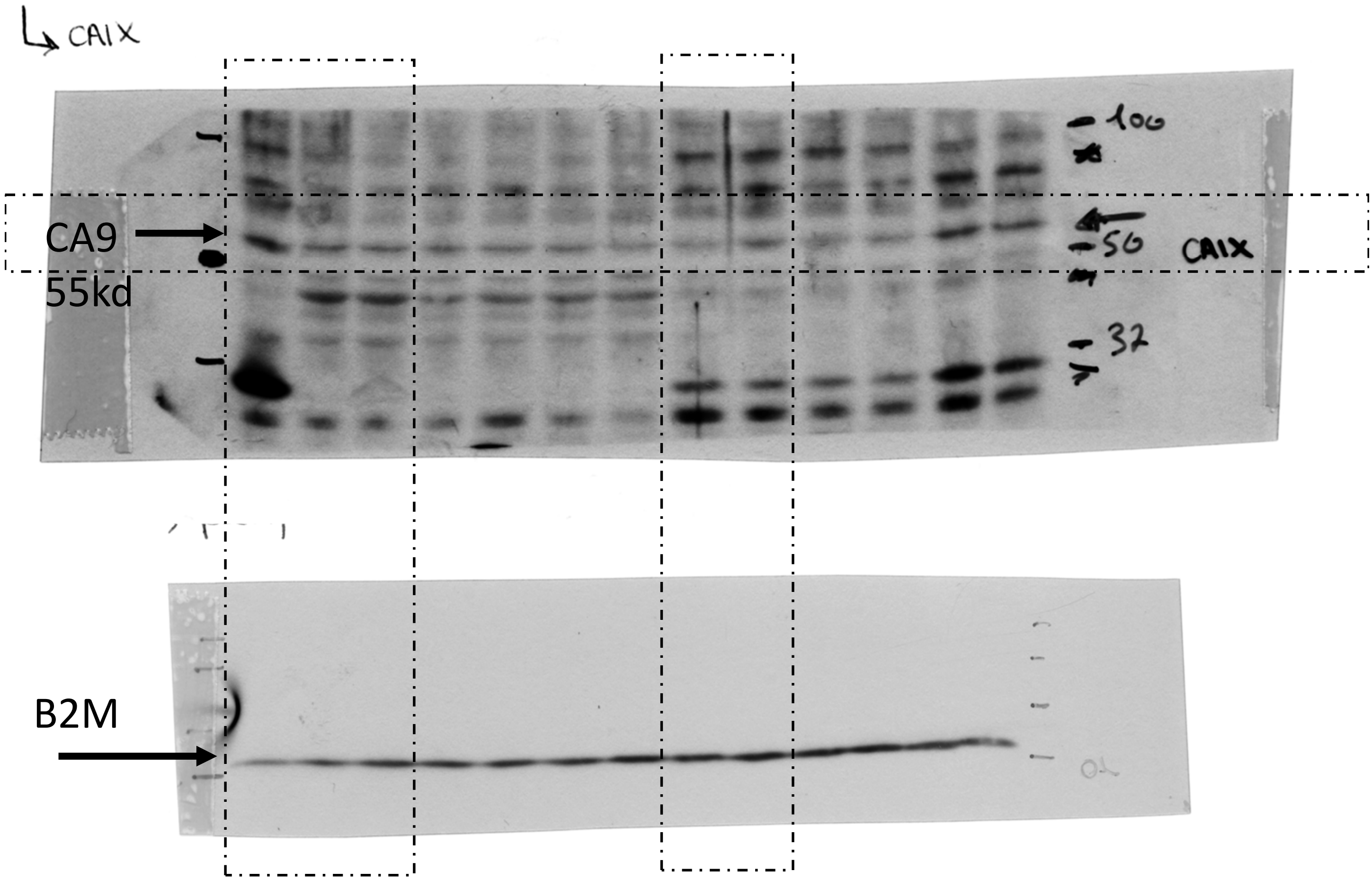

Figure 5A - SRC3

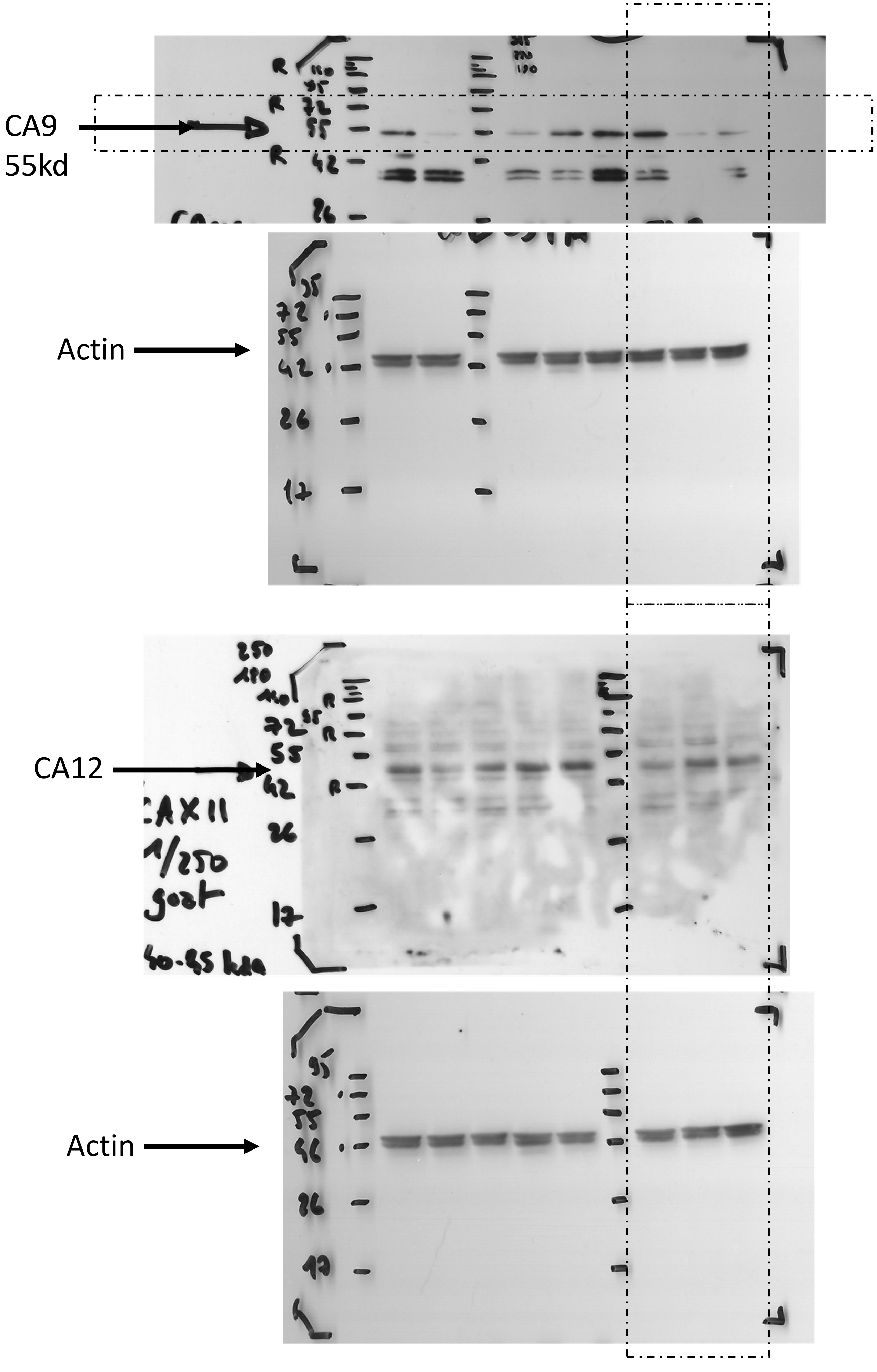

Figure 5B - SRC3

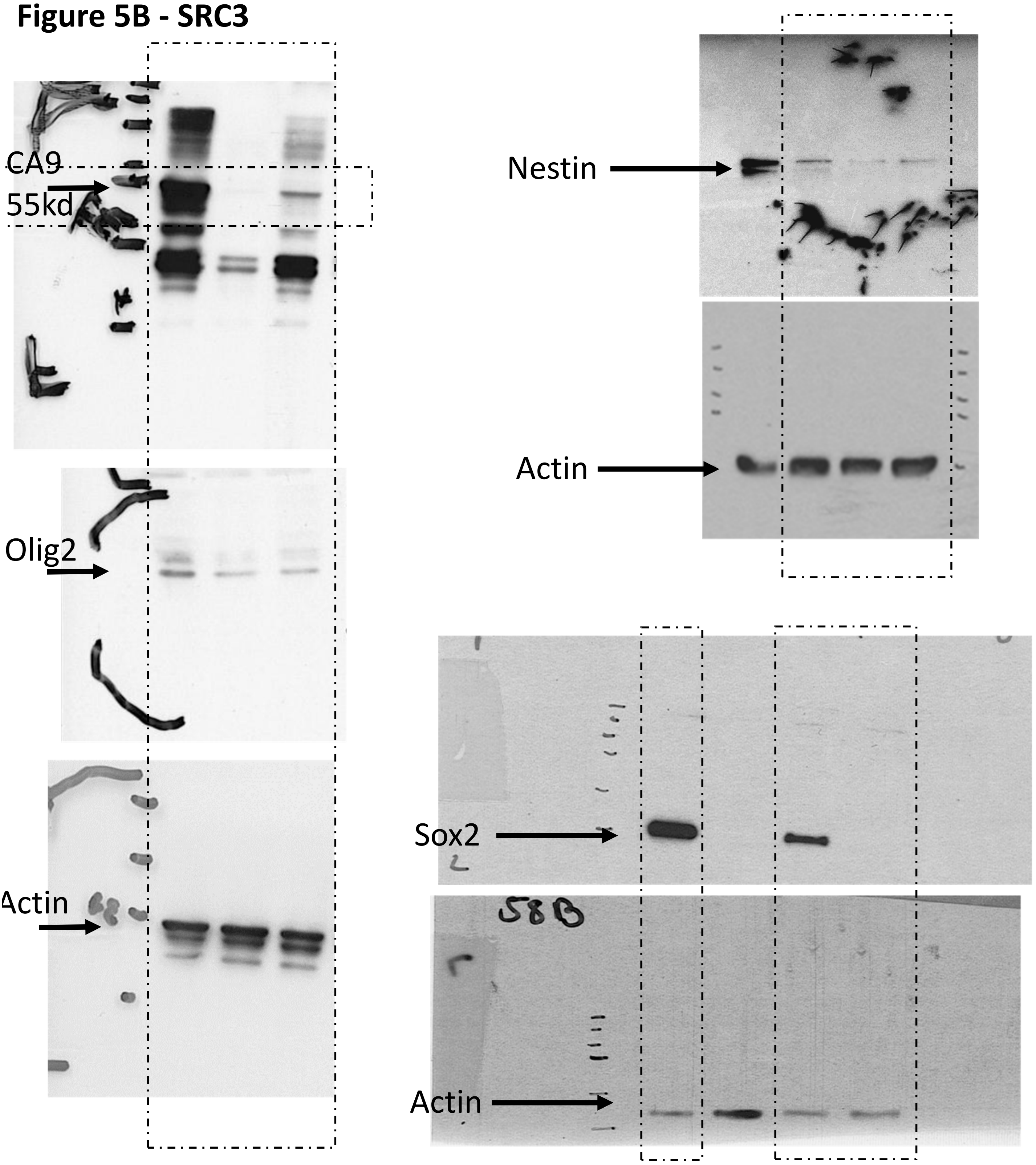

Figure 5B - D1

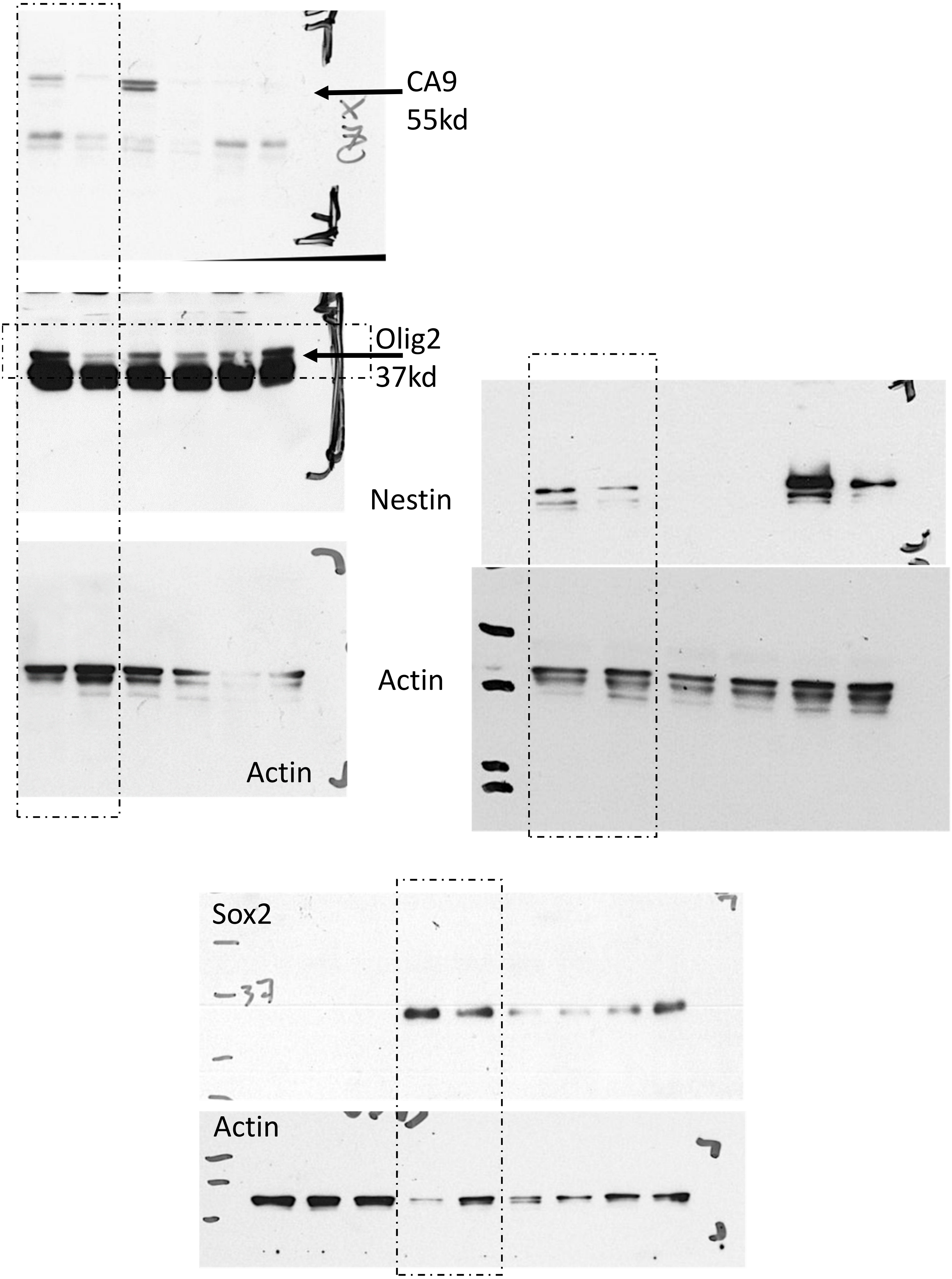

Figure 5 - G

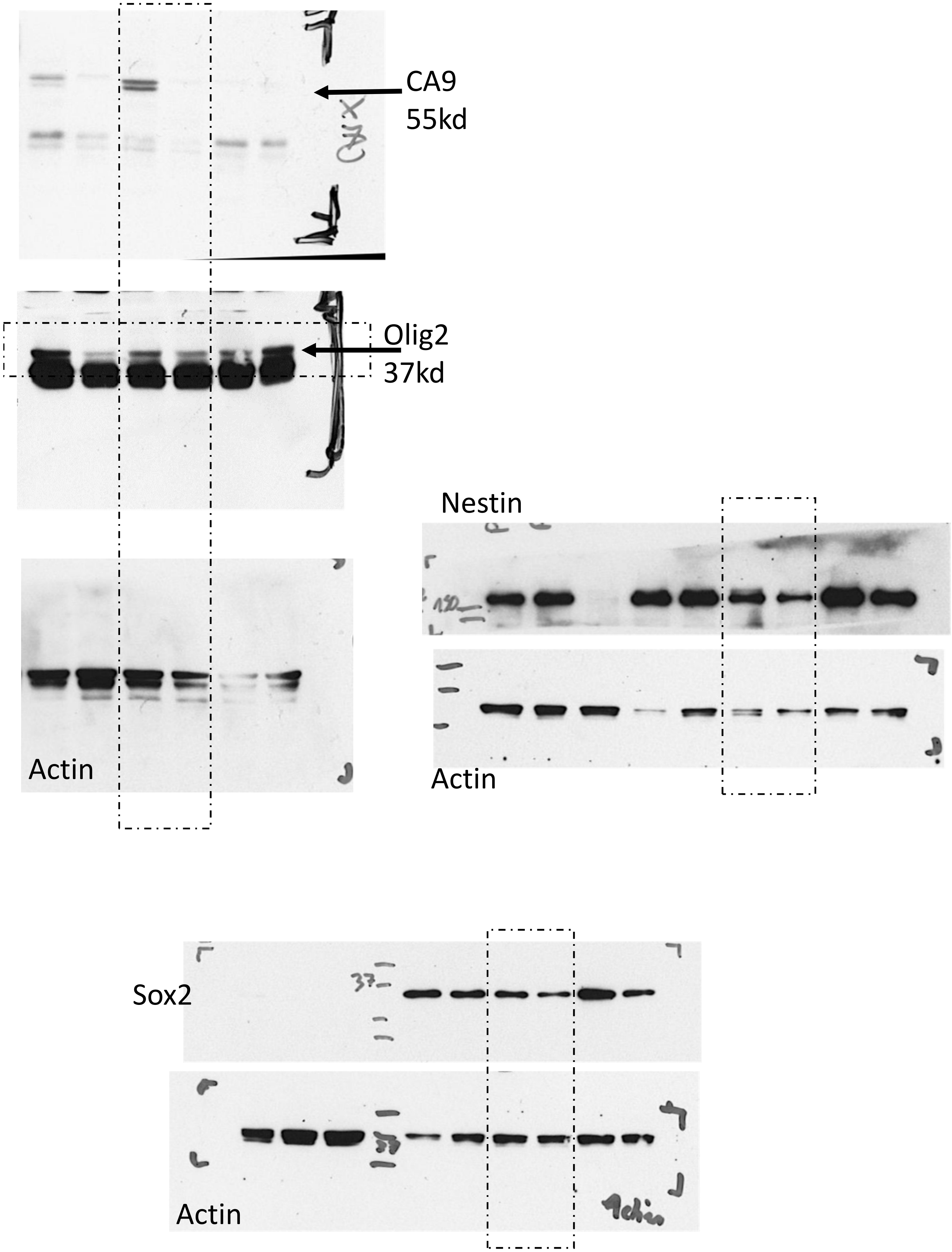

Figure 5 - I

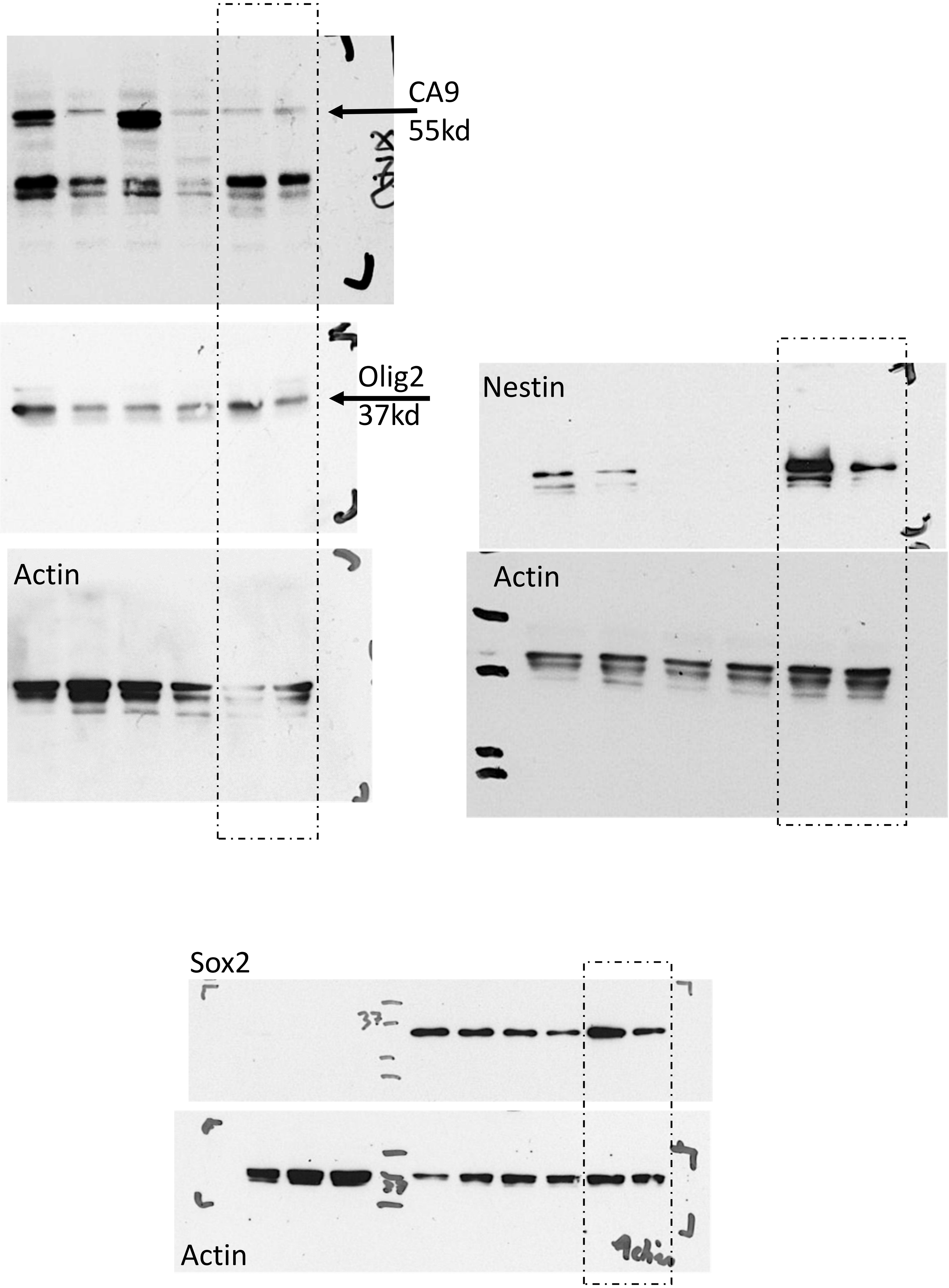

Figure 6B

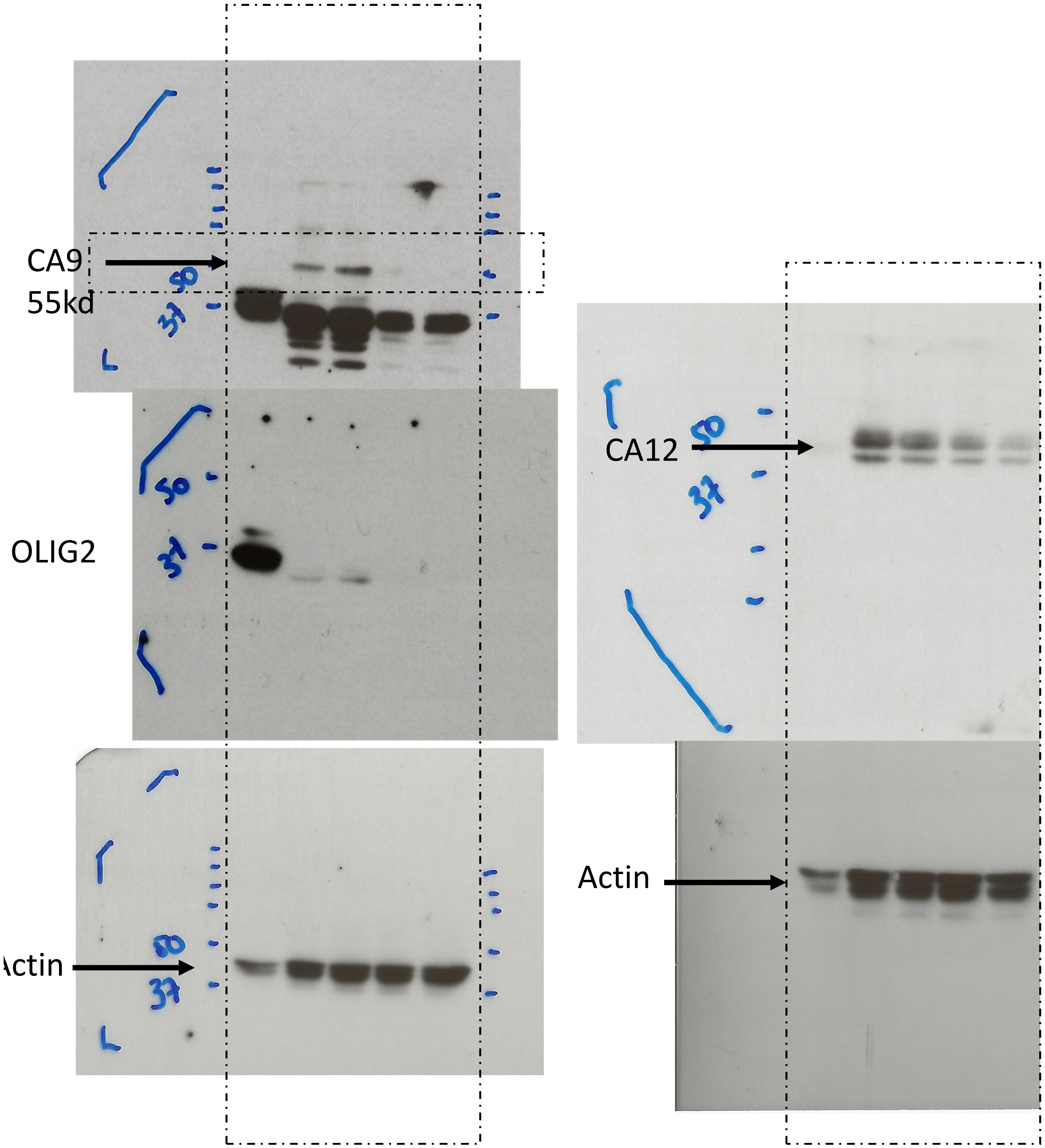

Supplement: Supplementary file 2 — Supplementary Material 2. [file 40478_2025_2161_MOESM2_ESM.pdf]
